# Towards Sustainable Recovery of Phenolics, Proteins, and Arabinoxylans from Brewer’s Spent Grain Through Deep Eutectic Solvents: Extraction Strategies, Structure–Function Relationships, and Food Biorefinery Perspective

**DOI:** 10.3390/foods15183173

**Published:** 2026-09-08

**Authors:** Mohammad Afzal Hossain, Benjamin T. Lobel, Andrew J. Currie, Costas Stathopoulos, Suwimol Chockchaisawasdee

**Affiliations:** 1 School of Medical, Molecular, and Forensic Sciences, Murdoch University, Murdoch 6150, Australia; afzal.hossain@murdoch.edu.au (M.A.H.); a.currie@murdoch.edu.au (A.J.C.); 2 Food Futures Institute, Food Innovation Precinct Western Australia, Nambeelup 6181, Australia; 3 Department of Food Engineering and Tea Technology, Shahjalal University of Science and Technology, Sylhet 3114, Bangladesh; 4 School of Mathematics, Statistics, Chemistry and Physics, Murdoch University, Murdoch 6150, Australia; b.t.lobel@murdoch.edu.au

**Keywords:** conventional extraction, DES recyclability, functionality, microwave-assisted extraction, structural preservation, sustainability, ultrasound-assisted extraction

## Abstract

Brewer’s spent grain (BSG) is the primary by-product of the brewing industry and a low-cost lignocellulosic resource rich in phenolics, proteins, and arabinoxylans (AXs). Conventional recovery methods using acids, alkalis, and organic solvents often involve energy-intensive processes, generate hazardous waste, and limit food-grade applications. This review critically examines the evolution of extraction methodologies for BSG bioactives, highlighting the potential of deep eutectic solvents (DES) as sustainable alternatives. Key factors such as solvent chemistry (polarity, pH, and water content) influence bioactives’ recovery and selectivity. Process intensification techniques such as ultrasound, microwave, and pressurised liquid extraction enhance efficiency by reducing extraction time and temperature. The review assesses how various processes modify the structure–function properties of BSG bioactives, including antioxidant activity, protein functionality, and rheological behaviour. A significant finding is that DES research has primarily focused on single compounds, while integrated DES biorefineries for comprehensive valorisation remain underexplored. Future research should therefore prioritise integrated process design that balances recovery, structural preservation, functionality, and sustainability to support scalable, near-zero-waste BSG valorisation for food and nutraceutical applications.

## 1. Introduction

Brewer’s spent grain (BSG) is a primary by-product generated in the beer industry, representing the insoluble lignocellulosic fraction of malted grain remaining after wort production [1]. BSG accounts for approximately 30% of the malted barley used in brewing, resulting in an estimated annual global production of 37–40 million tonnes [2]. It is a rich source of valuable nutrients, including proteins, phenolics, lipids, and carbohydrates [2]. BSG contains 25–30% protein, with hordeins (A, B, and C) making up over half of the total, followed by glutelins [3,4]. It is also a good source of phenolic compounds, including catechin, caffeic acid, ferulic acid, and *p*-coumaric acid [2]. Carbohydrates make up 60% of BSG, with dietary fibre accounting for half of this content. The fibre consists of approximately 28% lignin, 17% cellulose, and 25–30% hemicellulose [2,5]. The primary hemicelluloses are AXs, which have a β-(1 → 4)-linked xylopyranose backbone. Although AXs are sugars, they are classified as a dietary fibre because they resist hydrolysis by digestive enzymes [5,6].

BSG is frequently disposed of as waste or utilised for biogas production and animal feed. It possesses several important characteristics, including low cost, abundance, biodegradability, and compatibility with integrated biorefinery and food processing systems [7,8]. These attributes position BSG as a promising candidate for valorisation within a circular bioeconomy framework [9]. Consequently, BSG has been used in developing value-added products, including packaging materials, prebiotics, and functional food ingredients enriched with BSG-derived fibre, protein, and micronutrients [2,10]. The bioactive compounds in BSG are strongly associated with the cell wall matrix, necessitating the use of chemically harsh extraction methods, such as acids, alkalis, and organic solvents, to achieve efficient recovery [5,11]. The most common traditional method to recover these functional biomolecules from BSG is the use of organic solvents such as ethanol, methanol, acetone, etc. [12,13,14]. Nevertheless, growing awareness of their environmental burden and potential human health risks has shifted research focus toward the adoption of green, sustainable solvents and technologies in the food and pharmaceutical sectors [4,15,16,17]. Recent studies have identified deep eutectic solvents (DES) as environmentally friendly alternatives to traditional organic solvents for extracting bioactives from BSG [11,12,17,18].

A DES is a homogeneous mixture composed of one (or more) hydrogen-bond donor(s) (HBD) and a hydrogen-bond acceptor (HBA), combined at a defined molar ratio. This combination forms a joint superlattice, resulting in a eutectic melting temperature substantially lower than those of the individual components, primarily due to strong hydrogen bonding and charge delocalisation [19,20,21,22]. DESs are solvents that possess highly tunable physicochemical properties, including viscosity, density, polarity, and pH. By appropriately selecting the HBD and controlling water content and temperature, these properties can be tailored to enable efficient extraction of a wide range of biomolecules [11,23]. DESs have increasingly been used as green extraction media because of their ease of preparation, low cost, biodegradability, nonflammability, renewability, low toxicity, environmental compatibility, and effectiveness in recovering valuable bioactive components from BSG [17]. These include phenolic compounds with antioxidant properties, proteins with high nutritional value, and lignocellulosic fractions such as cellulose and hemicellulose-associated AXs [14,24]. Recent studies show that DESs offer improved extraction efficiency and selectivity compared to traditional solvents [11,12,14,24].

Despite the growing adoption of DESs as sustainable extraction media, conventional extraction techniques still dominate industrial practice and are associated with several challenges. These methods tend to be time-consuming, labour-intensive, economically inefficient, and energy-intensive [25]. To overcome these limitations, various advanced extraction technologies have been explored, including ultrasound, supercritical carbon dioxide extraction, and microwave-assisted extraction [26,27,28]. Among these techniques, ultrasound-assisted extraction has been reported to be highly effective for recovering bioactive compounds from BSG [24]. This effectiveness is attributed to its enhanced extraction efficiency, reduced processing time, lower energy consumption, and strong adherence to green chemistry principles [10,17,24]. A recent study by Paiva Santiago et al. found that a two-stage ultrasound-assisted extraction with DESs significantly increased phenolic recovery from BSG [24].

Most studies in the literature on the extraction of bioactive compounds such as phenolics, proteins, cellulose, hemicellulose, and AXs from BSG have focused on isolating a single component [11,12,29]. Despite the presence of multiple compounds with diverse properties and functional benefits in BSG, single-target extraction approaches are often economically inefficient and limit overall resource utilisation. Recent studies have increasingly explored integrated BSG valorisation strategies aimed at recovering multiple high-value components while improving process sustainability and reducing industrial waste. For instance, acid–alkali fractionation has been applied to recover proteins along with hemicellulose derivatives [30]. Subsequently, combined enzymatic and hydrothermal processing has demonstrated high recovery efficiencies: 65% for proteins, 91% for oligosaccharides, and 1.1 g gallic acid equivalent (GAE)/100 g dry BSG for phenolic compounds. This method also resulted in impurity-free oligosaccharides, highlighting the growing interest in multi-component recovery strategies for improving the sustainable utilisation of BSG [31].

However, to the best of our knowledge, no previous review has comprehensively evaluated traditional and DES-based extraction strategies for the integrated recovery of phenolic antioxidants, proteins, and AXs from BSG. The absence of a critical assessment of both single-target and multi-stage green biorefinery approaches highlights an important gap in the current literature and underscores the need for a more integrated valorisation framework. In addition, the effects of extraction processes on the structure–function properties of recovered bioactives remain insufficiently addressed. This is a critical gap because conventional, DES-based, and intensified extraction methods can modify phenolic release, stability, and antioxidant activity; protein unfolding, solubility, emulsifying and foaming properties; and arabinoxylan molecular weight, substitution pattern, rheology, and prebiotic potential. Accordingly, this review critically evaluates BSG extraction strategies reported primarily over the last decade, with emphasis on their relationships with structural preservation, functional performance, and DES selectivity. Based on this analysis, the review proposes future directions and sustainability-oriented solutions to advance integrated BSG biorefineries for the efficient recovery of high-value bioactives, thereby achieving near-zero-waste BSG valorisation.

## 2. Prospects of BSG as Sustainable and Functional Food Ingredients

One of the key steps in beer processing is wort production, in which carbohydrates are extracted from malted barley, leaving behind an insoluble lignocellulosic residue [32]. This solid residue, commonly known as BSG, is the most abundant by-product generated in the brewing industry. BSG accounts for approximately 85% of total brewery by-products and is composed mainly of barley seed coat, husk, pericarp, and endosperm [32,33]. Chemically, BSG is a lignocellulosic matrix rich in dietary fibre and protein. Its fibrous fraction consists mainly of cellulose, hemicellulose, and lignin, with minor components including residual starch, sugars, lipids, and minerals [2,34]. It also contains significant levels of bound phenolic acids, contributing to its antioxidant potential [35].

**Figure 1:**
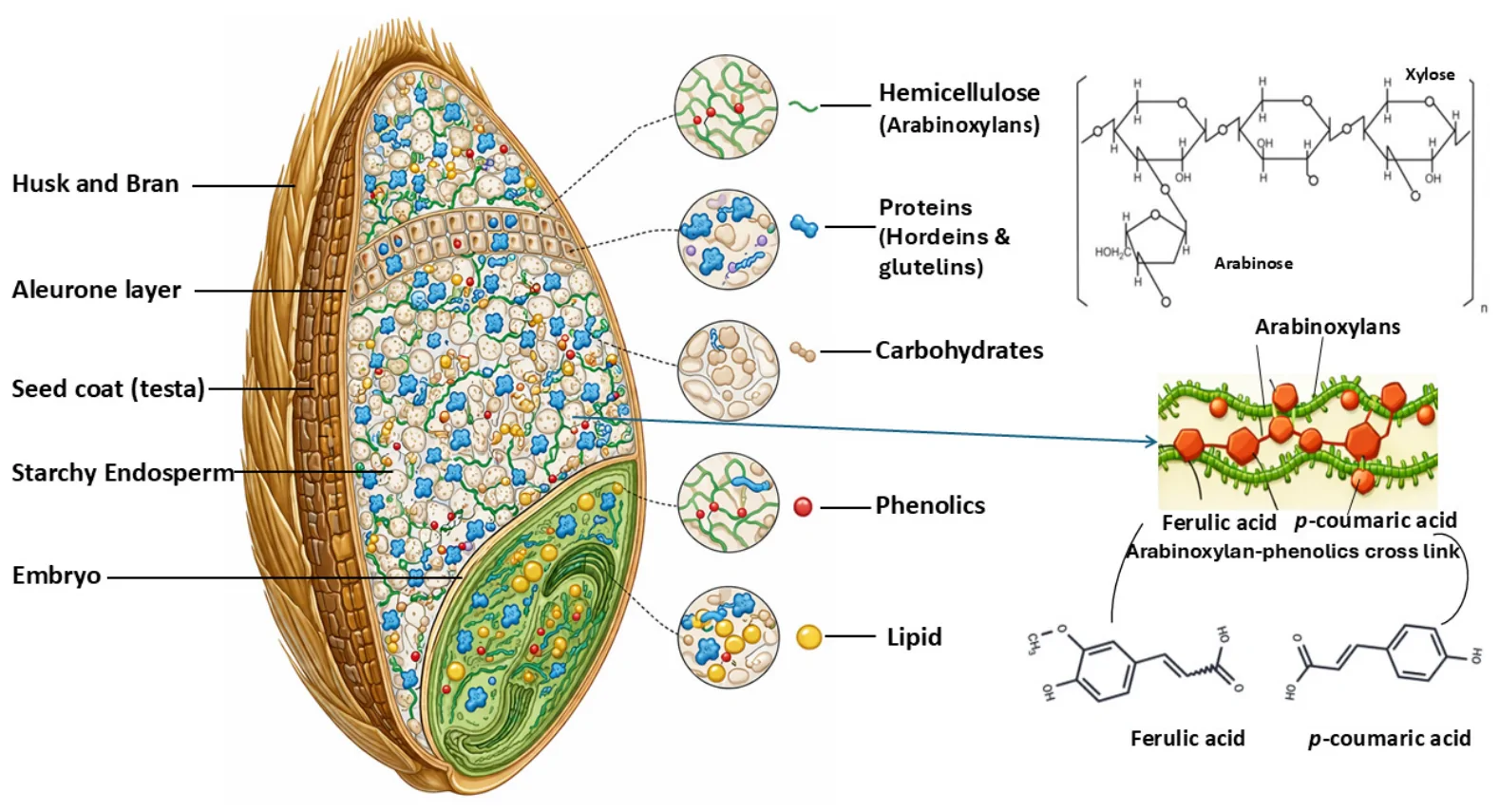
Schematic cross-section of a barley grain showing the spatial distribution of major macromolecules relevant to brewers’ spent grain.

BSG typically contains 70–80% moisture. On a dry basis, it comprises approximately 60–70% fibrous carbohydrates, 15–30% protein, 5–10% lipids, 2–5% minerals, and bioactive phytochemicals [4,5,34,36]. The fibre fraction is primarily composed of cellulose (~16–29%), hemicellulose (~20–40%), and lignin (~12–28%), with AXs representing the dominant hemicellulosic component [36,37]. In practice, composition varies with barley variety and brewing conditions but consistently reflects BSG as a fibre- and protein-rich material with notable phenolic content. Figure 1 illustrates the schematic structure and positions of BSG bioactives in an intact barley grain. In recent years, research has increasingly focused on the extraction of proteins, phenolic compounds, and AXs from BSG due to their relatively accessible recovery, high added-value potential, and suitability for food, nutraceutical, and biorefinery applications [34,37].

### 2.1. Phenolic Compounds

BSG is rich in cell wall-bound polyphenols, primarily hydroxycinnamic acids. According to Paiva Santiago et al. BSG may contain 57 flavonoids and 26 phenols, with ferulic acid and *p*-coumaric acid being the most abundant [24]. Sajib et al. reported approximately 1219 µg/g of ferulic acid and 489 µg/g of *p*-coumaric acid in dry BSG, respectively [38]. Other phenolics, such as syringic acid (78 µg/g) and catechin (29 µg/g), are present at much lower concentrations [38,39]. Over 80% of ferulic and *p*-coumaric acids in BSG are insoluble. They are bound to AXs and lignin via an ester bond in the grain husk (Figure 1), requiring alkaline or enzymatic hydrolysis for release [27]. These phenolic acids are potent antioxidants and bioactives and have been demonstrated to scavenge free radicals in vitro and attenuate oxidative stress in cellular and physiological systems. In their pure form, these compounds exhibit anti-inflammatory, anti-atherogenic, and anti-proliferative effects on cancer cells in vitro [10,27,40]. BSG-derived phenolic extracts have shown strong radical-scavenging capacity and protective effects, including in vitro prevention of oxidative DNA damage [27]. As a result, many studies focus on recovering these phenolics, positioning BSG as a promising source of natural antioxidants and functional ingredients [41,42,43,44].

### 2.2. Proteins

BSG contains a significant protein fraction, primarily composed of water-insoluble prolamins (specifically hordeins) and glutelins. Hordeins function as storage proteins, while glutelins serve as structural proteins in barley [45,46]. Laboratory methods have recovered BSG protein as concentrates or isolates [47,48,49]. Conventional extraction techniques include acid, alkali, and enzymatic extraction, whereas emerging methods utilise DES, with or without the application of processes such as ultrasound or microwave treatment [16,50]. These extracts exhibit broad molecular weight (M_w_) distributions, arising from both the intrinsic heterogeneity of hordein protein classes and process-induced proteolysis during malting and mashing, and demonstrate favourable techno-functional properties, including emulsifying, foaming, and gelling behaviours [16]. Consequently, BSG is considered a valuable plant protein source for both food and feed applications.

### 2.3. Arabinoxylans (AXs)

Hemicellulosic polysaccharides generally constitute approximately 20–40% of the dry weight of BSG, with more than 84% of these polysaccharides identified as AXs [6]. In raw BSG, AXs are largely insoluble because they are embedded within the lignocellulosic matrix of the husk, where they are cross-linked with lignin and phenolic acids through ester and ether bonds [36]. Structurally, AXs consist of a β-(1→4)-linked xylose backbone (Figure 1) substituted with arabinose residues [51]. Ferulate-mediated cross-linking, in which ferulic acid forms ester and diferulate bridges between AX chains and lignin, is a key structural feature that limits AX extractability by increasing cell wall rigidity and resistance to enzymatic degradation [52]. Therefore, pretreatment strategies such as alkaline, hydrothermal, or extrusion-assisted processes are necessary to disrupt these linkages and enhance enzymatic degradation for AX solubilisation [53,54]. Upon hydrolysis, AXs yield arabinoxylo-oligosaccharides (AXOS) and monosaccharides that demonstrate prebiotic activity and are fermentable by gut microbiota. BSG-derived AXs and AXOS have been shown to selectively stimulate beneficial bacteria, improve glycaemic response, serve as potential wall materials for encapsulation, and lower cholesterol levels, underscoring their significant functional food potential [53,54]. The high abundance, structural complexity, and bioactivity of BSG-derived AXs position them as a valuable target for valorisation in biorefinery applications.

Despite its availability, BSG remains underutilised. Traditionally, BSG serves as low-cost animal feed or is composted due to its wet and heterogeneous composition, which complicates storage [9]. Therefore, recent research has shifted focus toward extracting antioxidative phenolic compounds, thereby identifying higher-value applications [4,10]. Proper valorisation of BSG can reduce its environmental burden (e.g., methane emissions from landfilling) while supplying valuable biochemicals [24]. Achieving efficient and sustainable extraction of these compounds is a key goal in current BSG valorisation research.

## 3. Conventional and Emerging Extraction Approaches for BSG Bioactives

Various extraction methods have been utilised to recover proteins, phenolics and AXs from BSG, thereby transforming this brewing by-product from a low-cost feedstock into a higher-value product. Key approaches include solvent-based extraction, acid/alkaline hydrolysis, enzymatic digestion and subcritical water extraction [16,55]. Each method offers distinct advantages and disadvantages (Table 1).

### 3.1. Solvent (Solid–Liquid) Extraction

Solid–liquid extraction with conventional solvents is the simplest approach to recover BSG bioactives. This process involves soaking or refluxing BSG in solvents such as water, ethanol, methanol, and acetone to dissolve target compounds. This method has been widely used for polyphenols due to its low cost and ease of use [27]. However, conventional ethanol or acetone systems recover only limited amounts of phenolics from BSG because most compounds are bound to the fibre matrix. This is reflected in Zuorro et al., who extracted phenolics using ethanol and acetone and obtained 4.126 mg GAE/g after a four-stage, 4-h extraction with 60% (*v*/*v*) acetone–water, illustrating the typical low yields achieved with organic solvents [41]. Solvent extraction is even less effective for proteins and AXs. Most proteins in BSG do not dissolve in organic solvents, except for the prolamin fraction, hordein, which can be partly extracted with aqueous ethanol [47]. AX fibres also do not dissolve in organic solvents because of integrated cross-linking within the cell walls, unless they are further treated, such as by steam explosion, enzymatic, ultrasound, or microwave-assisted extraction [54]. In practice, extracting BSG with solvents often requires pretreatment or combined methods such as milling and alkaline or enzymatic hydrolysis to achieve better results [27,43]. The main problems with traditional solvent methods are long extraction times, sometimes up to 16 h, and the need for large amounts of flammable solvents [27]. These drawbacks make the solvent extraction process less sustainable. However, solvent extraction provides a baseline recovery of BSG antioxidants that can be improved upon by more efficient techniques [43].

### 3.2. Chemical Hydrolysis (Alkaline and Acid Extraction)

Chemical hydrolysis uses reagents to break down the complex cell wall of BSG, thereby releasing bioactive compounds that would otherwise remain trapped. Alkaline extraction is the most widely used chemical method for BSG valorisation because it cleaves ester linkages, such as ferulate–AXs bonds, thereby deconstructing the cell wall to release bound phenolics and make polysaccharides soluble [27,49,54,56]. This approach is widely recognised for significantly increasing yields at varying temperatures and treatment durations. For example, Bucci et al. found that repeated alkaline pretreatment of BSG with 0.5 M sodium hydroxide (NaOH) at 40 °C for 4 h each produced 5.5 g of ferulic acid (FA) and 0.6 g of *p*-coumaric acid per kg of BSG. These amounts of bioactives were lower than those from hydrothermal treatment (210 °C, 1 h) and higher than those from enzymatic treatment (cocktail of esterase and endo-xylanase, 25 °C, 4 h), used in the same study [43]. Alkaline treatment is also the standard for protein recovery. Silva et al. reported that alkaline treatment of BSG with NaOH yielded up to 87% of the total proteins at pH 11 and 60 °C over a 3 h extraction period [71]. Another study reported that a combination of defatting, delignification, and alkaline treatment with 0.01 M NaOH at pH 12.4, followed by three-times-repeated extraction, resulted in 45% protein extraction [49]. Mild alkaline conditions can also facilitate the liberation of AXs. Conventional caustic extraction methods have been shown to release approximately 65% of the AXs content from BSG when using 0.5 M NaOH at pH 11–12 [72]. Nonetheless, exposure to strong alkali can degrade sensitive molecules. For example, some phenolics may oxidise or form humins, and proteins may be partially hydrolysed, which reduces their functionality. In contrast, acid hydrolysis is not often used to extract intact bioactive compounds from BSG. Sometimes, acids such as dilute H_2_SO_4_ are used to break down hemicellulose into fermentable sugars for biofuel production [73].

Alkaline extraction of BSG faces criticism for its environmental, economic, and technical drawbacks. This method relies on strong caustic chemicals, which create a significant chemical footprint and CO_2_ emissions, challenging its credentials as a “green” process [74]. Managing and neutralising waste from these chemicals is expensive and labour-intensive, making large-scale use less practical [27,75]. Alkaline extraction also often takes several hours and involves multiple steps, such as pH adjustment and isoelectric precipitation for protein recovery, which adds to the processing time and energy needed [27]. Another major issue is that this method is not selective for BSG bioactives. The harsh conditions dissolve not only the desired proteins, phenolics, and AXs, but also unwanted lipids, lignin, and other sensitive compounds [75].

### 3.3. Enzymatic Extraction

Enzymatic approaches use cell-wall-degrading carbohydrases and proteases, such as α-amylase, xylanases, Alcalase, and cellulases, to break down hemicellulose and cellulose in BSG fibre and gently extract BSG bioactives [4,61]. Instead of harsh chemicals, enzymes specifically target polysaccharides and proteins, thereby producing smaller and soluble peptides. They also improve phenolic recovery by breaking covalent bonds within the cell wall, offering a green, selective extraction method [4]. Xylanase pretreatment cleaves AX chains, releasing ferulic acid, which is ester-linked to these chains. Previous studies have demonstrated significant improvements in ferulic acid yields from BSG using feruloyl esterases and xylanases [42,76]. Enzymatic extraction methods have shown promising results in increasing BSG protein yields, although yields varied across studies, ranging from 30% to 86% and averaging 40% to 50%, depending on the enzymes and conditions used, as summarised by Devnani et al. [4]. Connolly and co-workers demonstrated that the combination of carbohydrase, Alcalase, and protease achieved a 63% protein extraction yield, which was significantly higher than yields obtained through the alkaline (~59%, 0.11 M NaOH) extraction process [64]. Belardi et al. recovered 65% of the protein from wet BSG using Alcalase, a serine protease, at pH 8.5. This process also released phenolics, measured at 11 mg GAE/g of BSG, into the extract [31]. This finding demonstrates how enzymatic hydrolysis of the matrix can extract phenolic antioxidants together with protein. Enzyme-assisted extraction has also been shown to enhance phenolic recovery from BSG, with Crowley et al. reporting increased antioxidant activity and phenolic content resulting from the release of bound hydroxycinnamates compared with aqueous extraction [61]. However, the results of that study were inconsistent, and some enzyme treatments produced lower results than the control (water extraction, without enzymes, pH 7).

Enzymatic methods are usually milder than alkaline extraction. They work at moderate pH levels, often between 5 and 9, and at temperatures from 40 to 60 °C [16]. This helps preserve the bioactivity of compounds and avoids harsh chemical residues. These methods are also target-specific, such as using β-glucanase for β-glucans or xylanase for AXs, which can produce more selective fractions [16]. The primary challenges include high operational costs, time-intensive processes, and incomplete extraction. Enzymes are often expensive, and no single enzyme can hydrolyse all linkages in the complex BSG cell wall. Incomplete enzymatic digestion may leave some material unextracted. Consequently, enzymatic extractions require careful optimisation of parameters such as pH, temperature, reaction time, and enzyme dosage to achieve maximum efficacy [16,36]. In practice, enzymatic extraction is often combined with methods such as mild alkali treatment or mechanical disruption to maximise yields [16,42]. Nevertheless, enzyme-assisted strategies enhance protein extraction without the harsh conditions of chemical hydrolysis. These advantages make enzyme-assisted extraction an attractive and sustainable approach for BSG fractionation.

### 3.4. Subcritical Water Extraction

Subcritical water extraction uses heated water (120 to 200 °C) at high pressure to act as a solvent and catalyst that releases bioactive compounds. At these temperatures and pressures, water remains liquid, but its dielectric constant decreases, and concentrations of H^+^ and OH^−^ ions rise, resulting in a reactive hydrolytic medium. Simply put, hot compressed water can break glycosidic bonds and hydrolyse esters [77]. This technique is also known as hydrothermal treatment or autohydrolysis. It is considered an environmentally friendly way to deconstruct BSG because it only requires water. This method has demonstrated increased efficacy in extracting oligosaccharides and solubilising hemicelluloses. Hydrothermal autohydrolysis can achieve near-quantitative recovery of hemicellulose-derived oligosaccharides from BSG [60,67]. Belardi et al. [31] reported that a mild hydrothermal treatment at 150 °C for 60 min solubilised up to 91% of BSG’s carbohydrates as soluble oligosaccharides, with minimal formation of degradation products such as furfural or 5-hydroxymethylfurfural (HMF). The process utilises the acetyl groups naturally present in hemicelluloses, which, at high temperatures, generate acetic acid in situ and catalyse the autohydrolysis of the xylan backbone. In addition to oligosaccharides, subcritical water has been reported to extract phenolics and proteins. Alonso-Riaño et al. showed that in addition to breaking down hemicellulose, subcritical water (185 °C, 150 min) was able to extract 64 to 78% of proteins from BSG, matching the extraction efficiency of alkaline methods [60].

Since hydrothermal extraction does not use any corrosive chemicals, it has been considered a clean method that leaves no toxic residues and can produce food-grade extracts. It is also fairly fast because hot water reacts quickly [67,78]. Controlling the temperature and time is very important. If the conditions are too severe (i.e., prolonged time and high temperature), sugars and phenolics can break down into furans [67]; however, when optimised, the process can be highly selective. For example, Belardi et al. obtained AXs-rich oligosaccharides without any detectable furfural or HMF impurities by adjusting the hydrothermal conditions at 150 °C for 60 min [31]. One disadvantage of using subcritical water is that it requires special pressurised reactors, which can be energy-intensive to heat [78]. The process may also produce a complex mixture of oligosaccharides, monomers, proteins, and phenolics, so further separation may be needed to isolate pure components [60].

**Figure 2:**
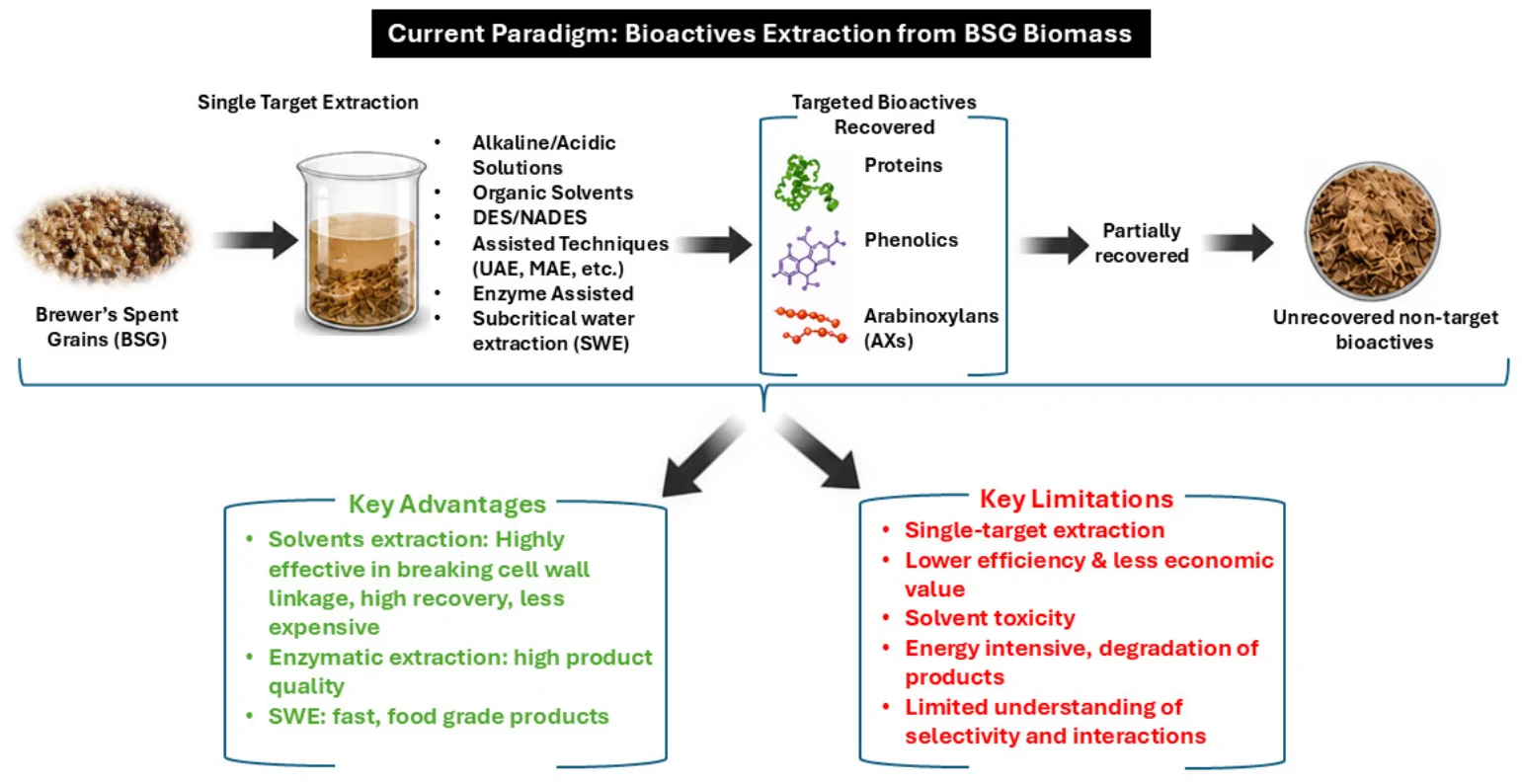
The current paradigm of bioactive extraction process from BSG has both advantages and disadvantages.

Although each of the aforementioned methods (Section 3.1, Section 3.2, Section 3.3 and Section 3.4) has achieved significant success in extracting bioactives from BSG, they also have disadvantages (Figure 2). The limitations of these methods underscore the need for safer, tunable, and more sustainable solvent platforms. This has accelerated interest in “designer solvents,” particularly DESs, which offer low toxicity, high tunability, and strong solvating power for diverse biomolecules [17].

## 4. Deep Eutectic Solvents: Properties and State-of-the-Art in DES Extraction of Bioactives from BSG Biomass

Deep Eutectic Solvents (DES) are novel designer solvents formed by mixing at least two components, typically a hydrogen bond acceptor (HBA) and a hydrogen bond donor (HBD), in a defined molar ratio [79,80]. Their markedly reduced melting points arise from strong hydrogen-bonding interactions between the components, which disrupt the crystal lattice of the pure substances and generate a liquid phase at temperatures far below those of the individual constituents [79,81]. A widely used HBA is choline chloride (ChCl), whose chloride anion (Cl^−^) forms extensive hydrogen-bond networks with HBDs such as polyols, organic acids, and amides. Abbott and his team demonstrated this principle with the classic choline chloride–urea system: combining ChCl (HBA) and urea (HBD) in a 1:2 molar ratio yields a room-temperature liquid, despite both components being solids with high melting points [21]. When composed of natural, non-toxic constituents, DESs are often described as “green” or “bio-based” solvents.

**Figure 3:**
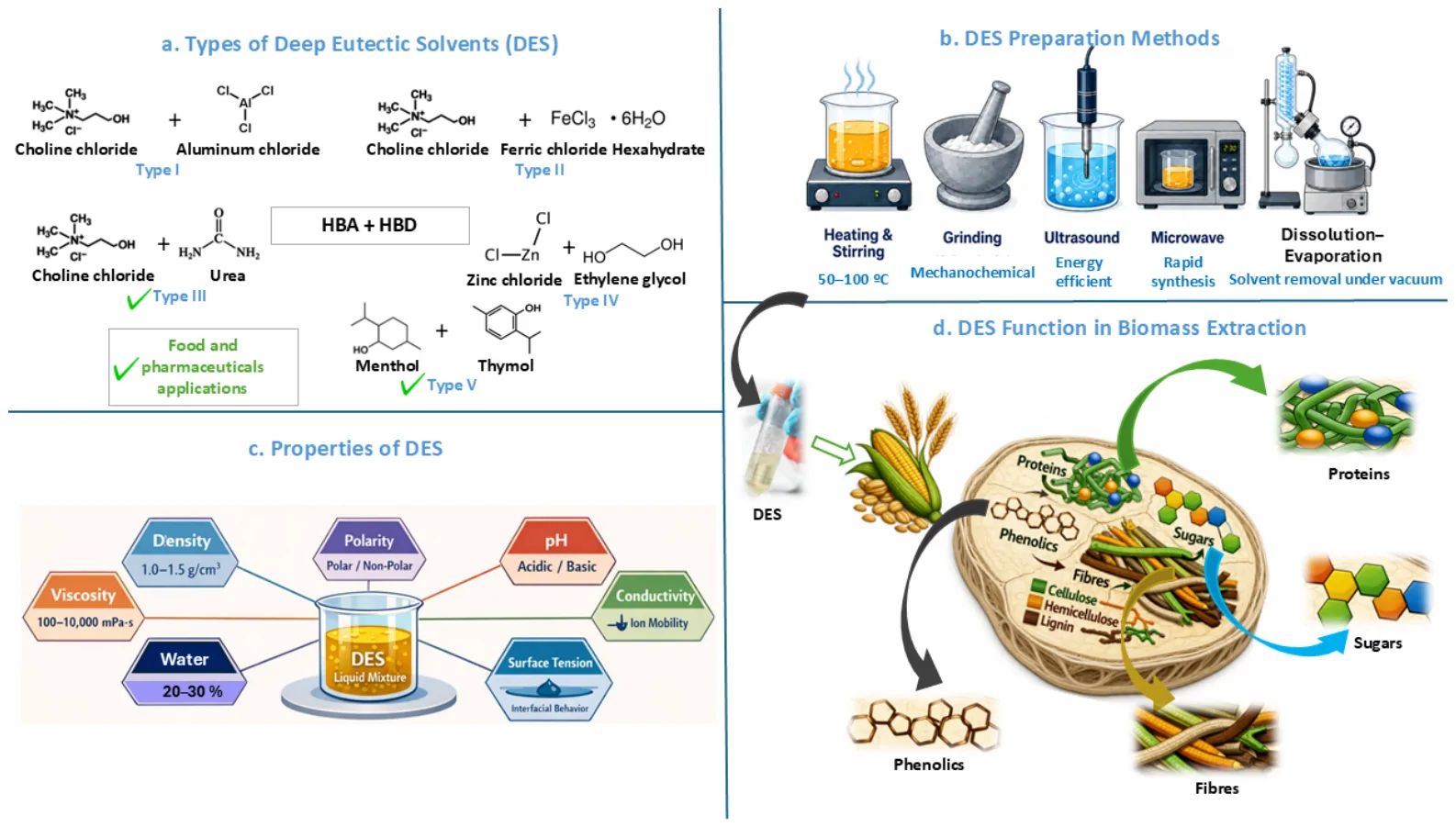
Structure–preparation–property–function relationship of deep eutectic solvents (DES) in biomass valorisation. (**a**) DES are formed through hydrogen-bonding interactions between HBA and HBD, resulting in eutectic mixtures. (**b**) Their preparation methods influence physicochemical consistency. (**c**) The tunable properties of DES govern their interaction with biomass matrices, and (**d**) DES properties determine extraction efficiency and selectivity for target bioactives.

DESs are classified based on the nature of their constituents and the types of interactions involved. Deep eutectic solvents (DES) are generally represented by the formula Cat^+^X^−^zY, where Cat^+^ denotes a cation, typically ammonium, phosphonium, or sulfonium; X^−^ corresponds to a Lewis base, most commonly a halide ion; and z indicates the number of Y molecules interacting with the anion. The most widely accepted classification, originally proposed by Smith et al. and subsequently expanded, divides DESs into five main types (I–V) [19,82]. Classes III and V have been widely investigated for extracting bioactives from food and biomass [19,81]. The most common (Type III) are prepared from an organic salt (e.g., chloride, betaine, proline) as HBA and a non-ionic HBD (e.g., urea, glycerol, lactic acid). Class V eutectics are formed solely from two non-ionic molecular compounds [79]. DESs can be readily prepared by mixing HBAs and HBDs at a specified molar ratio, followed by heating or grinding, as illustrated in Figure 3 [79].

### 4.1. Properties of Deep Eutectic Solvents (DES)

DESs possess distinct properties that are governed by the nature and composition of the HBA and HBD used. The unique performance of DESs in biomass valorisation and other applications is rooted in their distinctive physicochemical properties, which are highly tunable via component selection, molar ratio, and water content [83]. The most critical physicochemical properties of DESs include phase behaviour, viscosity, density, pH, water content, polarity, and surface tension (Figure 3). The combination and molar ratio of HBA and HBD directly influence the phase behaviour, density, and polarity [84]. For example, a DES prepared from choline chloride and D-fructose (1:1) exhibits a greater density than that prepared from choline chloride and n-butyric acid (1:2), which is attributed to the larger structural cavity in n-butyric acid [85]. Deep eutectic solvents exhibit densities greater than water, usually ranging from 1.0 to 1.5 g/cm^3^ [85]. Adding water reduces the density of DES. Since water acts as an HBD, when it is added to the DES, it competes with DES for lignin interactions, thereby reducing the DES’s extraction functionality [86]. Assessing DES polarity is crucial for optimising extraction efficiency, as it directly affects the solvent’s solubility and selectivity for lignocellulosic components [87].

DESs are generally characterised by high viscosities at room temperature, typically 100–10,000 mPa·s, which are much higher than those of conventional organic solvents (0.2 to 10 mPa·s) [81]. Viscosity significantly influences mass transfer, solute diffusion, and process efficiency during extraction and pretreatment operations [83]. For example, choline chloride: glycerol (1:2) has a high viscosity of approximately 1200 mPa·s in the absence of water [81], primarily due to the extensive hydrogen-bonded supramolecular network between the HBA and HBD, which restricts molecular mobility and reduces free volume [84]. For instance, 30% water reduces the viscosity of choline chloride: glycerol (1:2) from 1200 to approximately 270 mPa·s. Similarly, heating from 25 °C to 80 °C can increase metal solvation rates by more than tenfold due to viscosity reduction [81]. Although high viscosity can benefit the stabilisation of sensitive biomolecules and reduce volatility, it presents considerable challenges for large-scale processing. Elevated viscosity increases energy requirements for mixing and pumping and restricts mass and heat transfer [88].

Acidic DESs are effective for delignification and hydrolysis of hemicellulose, whereas basic DESs, though less common, can facilitate ether bond cleavage in lignin [88]. Water acts as both a common impurity and a deliberate additive in DES systems, significantly influencing DES properties. Small amounts of water integrate into the hydrogen-bond network without “breaking” the DES, often improving mass transfer [89]. However, water concentrations exceeding 30–40% convert the DES into an aqueous solution rather than maintaining a true eutectic mixture [81]. Optimising water concentration is essential to reduce viscosity while preserving extraction selectivity and solvent stability [89]. Recent studies have also considered water as a stoichiometric component of DESs or low-melting mixtures (LMMs), rather than reporting it solely as a mass percentage, because water participates in the hydrogen-bond network. Aniskevich et al. [90] formulated lactic-acid-based mixtures as [ChCl][lactic acid]_x_[water]_y_ and [betaine][lactic acid]_x_[water]_y_ (where, x = 1, 2, or 10 and y = 1, 3, or 5) and demonstrated that both HBA identity and water stoichiometry affected viscosity and extraction behaviour, with a betaine:lactic acid:water mixture (1:10:5) showing the best extraction performance under optimised conditions. More recently, this stoichiometric approach was extended to ChCl–organic acid–water mixtures, with ChCl:citric acid:water (1:1:16) identified as an effective composition for phenolic extraction [91]. Surface tension governs the ability of DESs to wet biomass particles and penetrate plant cell walls. However, in DES systems, surface tension and viscosity largely reflect the strength of intermolecular interactions, particularly hydrogen bonding. Thus, weakening of hydrogen bonding generally lowers both surface tension and viscosity, which may also reduce solvent selectivity for target bioactives [92].

In addition to commonly reported physicochemical parameters, properties such as refractive index, thermal conductivity, and electrical conductivity are increasingly being considered when selecting DESs as extraction media [83]. The refractive index (RI) serves as a rapid indicator of solvent structural integrity and water uptake, whereas thermal conductivity affects heat transfer efficiency during extraction [93,94]. Variations in RI often indicate changes in DES composition, molar ratio, or water content, thereby providing indirect information about solvent structure and stability [93]. Thermal conductivity governs the rate and uniformity of heat transfer within the solvent–biomass system and therefore indirectly influences extraction kinetics [83]. Electrical conductivity, which reflects ion mobility and the degree of hydrogen-bond network disruption, also influences mass-transfer dynamics and process monitoring [83]. Although these parameters receive less attention than viscosity or polarity, they contribute to a more comprehensive evaluation of DES suitability for biomass extraction applications. Collectively, HBA/HBD composition governs DES acidity, polarity, and hydrogen-bonding interactions, while water content and temperature strongly influence viscosity and mass transfer. Consequently, these interdependent properties determine solute–solvent affinity and extraction selectivity rather than acting independently.

### 4.2. State-of-the-Art in DES Extraction of Bioactives from BSG Biomass

DESs have emerged as one of the most promising solvent platforms for the selective and sustainable extraction of phenolics and proteins from BSG. Although specific information on the extraction of AXs from BSG is limited, the physicochemical properties of DESs indicate potential for effective AX extraction from BSG. The tunability, low volatility, high extraction capacity, and potential food-grade compatibility make them attractive alternatives to aqueous alcohols, alkaline solutions, and enzymatic extractions as outlined in Section 3. Recent publications demonstrate rapid methodological advances, although challenges remain in extraction efficiency, viscosity management, and downstream recovery. Only a few studies have employed DES-based exclusive extraction of bioactive compounds from BSG; most have combined DES with intensification processes, which will be detailed in Section 5.

#### 4.2.1. Phenolic Compounds

Among phenolic compounds, ferulic acid, *p*-coumaric acid, and bound phenolic esters are the most targeted BSG bioactives. Recent studies show that HBA-rich DESs, especially choline chloride (ChCl) combined with organic acids such as lactic, citric, or malic acid, or alcohols like glycerol, significantly enhance the solubilisation of both free and bound phenolics [95]. DESs have demonstrated superior phenolic recovery from BSG compared to conventional solvents. For instance, Bleus et al. [96] reported that malic acid–ChCl DESs achieved substantially higher phenolic yields (78 μg/g) than acetone–water systems (~5 μg/g), attributed to improved disruption of lignin–carbohydrate complexes and enhanced cleavage of ester bonds. Similarly, Masiello et al. [97] demonstrated that optimising HBA–HBD combinations enhanced extraction performance, with a ChCl:lactic acid DES (30% water) yielding 2.86 mg GAE/g dry BSG, representing an approximate 15% increase compared to acetone–water (2.48 mg GAE/g dry BSG) and outperforming methanol–water systems (1.81 mg GAE/g dry BSG). Key BSG phenolics, including ferulic and *p*-coumaric acids, were recovered in these extracts. In another study, ChCl: malic acid (1:1) yielded TPC of 23 mg GAE/g dry BSG, slightly less than the 26 mg GAE/g dry BSG obtained from NaOH extraction [11].

The high polarity and hydrogen-bonding capacity of DESs enhance phenolic solubility and protect these compounds from oxidation, allowing highly polar phenolics to leach first and remain stabilised in the solvent [17]. DES methods have achieved high yields and quality of BSG phenolics, although most studies have employed microwave or ultrasound to accelerate extraction (Section 5). Even without such techniques, DESs with optimised compositions can surpass alcohols in phenolic yield [97]. However, direct benchmarking against aqueous alcohol, enzymatic, alkaline, and hydrothermal extraction should include not only total phenolic yield but also phenolic profiles, antioxidant stability, solvent consumption, extraction time, energy demand, solvent recyclability, and effects on the residual protein and arabinoxylan fractions.

While extracting BSG phenolics, high viscosity remains a major practical problem for many DESs, especially those made from organic acids. Viscous solvents slow down mass transfer; to overcome this, 20–40% water (*v*/*v*) is often added to the formulation to improve mass transfer. Unfortunately, this dilution can lower selectivity [24]. Another challenge is recovering phenolics from the DES after extraction. Anti-solvent precipitation and resin adsorption are effective techniques for phenol recovery, but their high energy demand, mainly from solvent recovery, resin regeneration, and product drying, limits their optimisation for large-scale use [27].

Recent work has recommended moving toward low-viscosity NADESs, creating tailored DESs with controlled pH, and developing smarter recovery methods that reduce solvent loss and enhance process sustainability [81]. Overall, DESs represent a promising platform for recovering BSG phenolics, but their superiority over established extraction methods remains dependent on solvent formulation, target compounds, process severity, and downstream recovery performance.

#### 4.2.2. Proteins

Protein extraction from BSG usually requires harsher conditions, so BSG proteins have traditionally been extracted with alkaline solvents [4]. Although alkaline extraction yields better results, there is a risk of denaturation and the Maillard reaction, which can impact protein functionality. DES can offer a milder alternative by selectively disrupting protein–polysaccharide interactions. DES-based protein extraction remains underdeveloped compared with phenolic extraction.

In a 2017 study, it was found that sodium acetate:urea (1:2) extracts 51% of BSG protein, whereas NaOH extraction achieved only 19% [98]. In a recent study, Hadinoto et al. used ChCl/trehalose NADES (3:1) to extract proteins under mild conditions (60 °C, 60 min) with the solid:solvent ratio of 1:15. They reported a 42% extraction yield versus a 34% yield using alkaline NaOH solution (pH 10). The NADES and alkaline-extracted proteins had similar molecular weights and amino acid profiles, but the NADES fraction showed greater thermal stability and better emulsifying and foaming functionality [50]. In both cases, DES-based extraction matched or exceeded conventional alkaline extraction in yield and quality, while operating at neutral or mildly basic pH. These findings indicate that DES can break hydrogen bonds in the protein-fibre matrix, releasing proteins without denaturing them, in contrast to strong alkali or organic solvents [50,98]. Although most studies focus on other biomass streams, few BSG-specific studies indicate that DESs can extract 20–40% of total protein under mild conditions (pH < 6, T < 60 °C), outperforming water and matching alkaline extraction while improving functional quality.

The main advantages of DES extraction, including mild conditions, reduced denaturation, and potential food-grade compatibility, are offset by the limited solubility of high M_w_ hordeins and glutelins, which may be improved through strategic tuning of DES composition and properties, and process optimisation [50,98]. Overall, the available evidence indicates that DESs can match or exceed conventional alkaline extraction under selected conditions while providing better preservation of protein functionality. Thus, the value of DESs should not be assessed solely by maximum extraction yield. Their potential lies in balancing protein recovery with reduced chemical severity, lower risk of structural damage, and the possibility of using food-compatible solvent components. However, further BSG-specific studies using standardised recovery metrics, direct process comparisons, solvent-recycling assessments, and detailed structural and functional characterisation are required before their industrial and food-grade applicability can be established.

#### 4.2.3. Arabinoxylans (AXs)

Brewer’s spent grain contains a large fibre fraction, approximately 25% of which is AXs [27]. Despite this, targeted DES recovery of AXs from BSG remains rare. In principle, DESs, especially those based on ChCl, are known to disrupt lignocellulose and lignin, enhancing polysaccharide accessibility. For BSG, strong alkali is typically used to cleave ferulate cross-links and solubilise AXs [51]. A few preliminary reports suggest that DES pretreatment can swell or partially dissolve BSG fibre, but AX yields have not been systematically quantified. Literature reviews note that DES extraction for BSG has not been extensively investigated, underscoring the nascent state of this application [17]. Thus, while DES chemistry promises a green route to recover AXs by selectively solubilising lignin and hemicellulose, more research is needed to establish protocols and yields for BSG fibres.

Hypothetically, DESs may offer a distinct advantage by combining solvent-mediated lignocellulose disruption with the ability to tune polarity, acidity, hydrogen-bonding capacity, and water content. Such flexibility could, in principle, enable selective weakening of lignin–carbohydrate and polysaccharide–protein interactions while limiting excessive AXs depolymerisation. However, this proposed advantage remains largely conceptual for BSG because direct comparisons between DES and conventional AXs extraction under equivalent conditions are not yet available. In particular, studies are needed to distinguish among total carbohydrate solubilisation, AXs extraction yield, AXs purity, molecular weight distribution, arabinose-to-xylose ratio, ferulic acid retention, and functional properties. Without these measurements, it is difficult to determine whether DES treatment recovers structurally intact AXs, generates lower-molecular-weight arabinoxylan oligomers, or simply increases non-selective fibre solubilisation.

In summary, DES extraction of BSG bioactives is at a promising but early stage. Notable successes have been achieved in phenolics and proteins, including improved yields, purity, and functional quality [50,81]. Addressing high viscosity and ensuring scalability will be key to making DES a practical part of sustainable BSG valorisation [81]. It is important to note that most existing studies remain confined to laboratory-scale investigations [17], thereby limiting current understanding of process scalability and industrial feasibility. Future work should focus on continuous processing, solvent regeneration, and evaluation of techno-economic and environmental performance. To date, specific DES processes for BSG AXs have remained largely unexplored, offering an open research opportunity.

## 5. Emerging Physical Intensification Techniques to Extract BSG Bioactives

**Figure 4:**
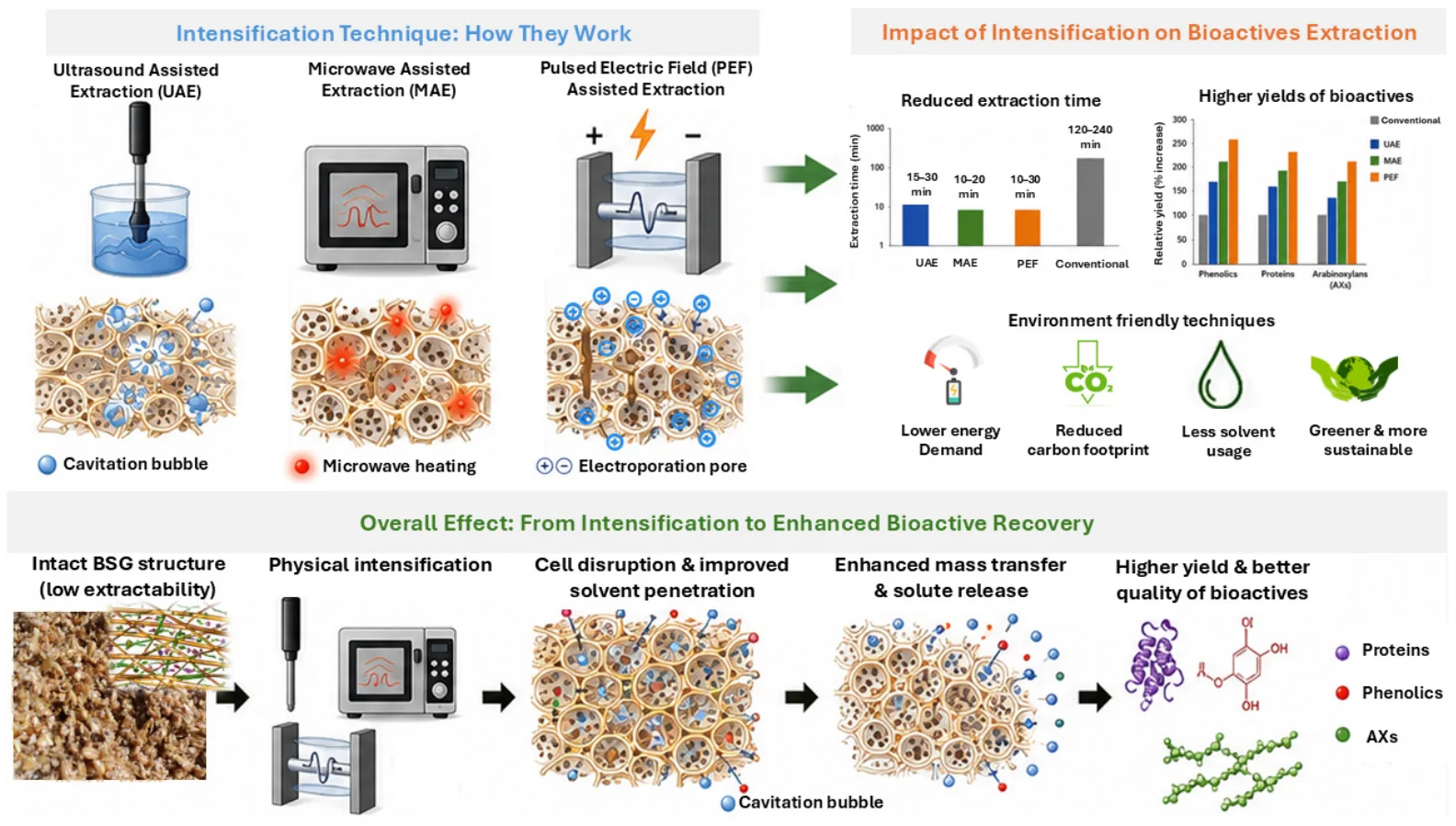
Schematic illustration of physical intensification techniques used to enhance the extraction of bioactive compounds from BSG.

The valorisation of BSG as a source of high-value biomolecules has progressed significantly through the development of both traditional and green extraction media. Nevertheless, the inherent resistance of the BSG lignocellulosic matrix often limits extraction efficiency, selectivity, and scalability. To address these challenges, various physical intensification techniques, including ultrasound-assisted extraction (UAE), microwave-assisted extraction (MAE), pulsed electric fields (PEF), and other emerging technologies, have been investigated in combination with DES-based and other extraction methods. As shown in Figure 4, these techniques enhance the recovery of phenolics, proteins, and AXs by disrupting the lignocellulosic matrix and improving mass transfer. Compared with conventional extraction, these techniques increase extraction yield while reducing processing time, energy demand, and environmental impact (Figure 4). This section provides a critical evaluation of research from the past decade on integrating these physical intensification methods with other approaches for extracting phenolics, proteins, and AXs from BSG, emphasising key findings, limitations, and future research directions as outlined in Table 2.

### 5.1. Ultrasound-Assisted Extraction (UAE)

Ultrasound-assisted extraction (UAE) uses acoustic cavitation to disrupt plant cell walls, enhancing solvent penetration and mass transfer [99]. When combined with other extraction methods, UAE has shown notable improvements in extracting phenolic compounds from BSG. Yu et al. studied protein recovery from BSG using Alcalase enzymatic hydrolysis under varying enzyme loadings (1–40 µL/g) and incubation times (0.25–24 h), with and without ultrasound (227.5 W/L, 10 min) pretreatment [100]. They found that ultrasound-assisted enzymatic extraction boosted protein separation efficiency to about 70%, cut enzyme use by around 73%, and shortened incubation time by about 56%. It also produced highly soluble hydrolysates (under 15 kDa) rich in glutamic acid and proline. The study did not examine the functional bioactivities of these hydrolysates beyond their solubility and basic characteristics [100]. In a recent study, Paiva Santiago et al. applied 30 °C ultrasound with choline chloride-based DES (ChCl:oxalic or lactic acid) to BSG, finding initial phenolic yields of only ~2 µg GAE/g of BSG extract. A two-step process (hexane or ethanol pre-extraction to remove lipids/sugars, followed by ultrasound/DES) raised phenolic yield to ~5 µg GAE/g of BSG extract [24]. Ultrasound-assisted extraction has recently been extended to the recovery of AXs from BSG. Liu et al. demonstrated that UAE (400 W, 60% duty cycle) increased AXs yield by 52.4% compared to traditional alkaline extraction (1.5 M NaOH), while reducing extraction time from 4 h to only 15 min. The process also resulted in AXs with lower M_w_ and particle size, improved solubility, and enhanced emulsifying and rheological properties [101]. These physicochemical improvements make low M_w_ AXs ideal for prebiotic applications and functional food ingredients [102].

Despite these promising results, ultrasound often requires high energy input, may denature proteins at high power, and typically focuses on a single fraction. Additionally, the decrease in M_w_ and viscosity of AXs may impair their ability to form stable hydrogels and reduce their efficacy in encapsulation applications, as lower M_w_ fractions typically yield weaker networks [103]. In the aforementioned studies, only phenolics or proteins were targeted, leaving sugars, lignin, and other nutrients largely in residues. In practice, this produces a “secondary waste” containing unextracted bioactives. These results suggest that single-step UAE recovers one component efficiently but at the expense of others, underscoring the need for follow-up extraction of the remaining biomass.

**Table 2:** Summary of BSG bioactives extraction combined with emerging intensification process.

Target Compound(s)	Intensification Technique	Main Extraction Solvents	Key Parameters	Yields/Results	Key Findings/ Limitations	References
Phenolic Compounds	Ultrasound-Assisted Extraction (UAE)	DES	-DES: ChCl:oxalic acid or ChCl:lactic acid; -US: 35 kHz, 100 W, 30–60 min; -DES molar ratio 1:1–2; -SLR = 1:10 (*w*/*v*) -Pre-washing with ethanol/hexane	-Initial yields 0.002 mg GAE/g BSG (*w*/*v*) (ChCl:OA, ChCl:LA); -After hexane or ethanol pretreatment up to 0.00516 mg GAE/g of BSG (*w*/*v*)	-US-DES showed highest phenolic recovery among tested solvents, but absolute yields remained low. -Selectivity depends on cleanup step; -Risk of phenolic degradation with prolonged sonication; -Only phenolics were recovered, protein/sugars remain in waste.	[24]
Phenolic Compounds	Pulsed Electric Field (PEF pre-treatment)	Solvent extraction (ethanol and methanol)	-PEF optimum conditions were electric field strength 2.5 kV/cm, frequency, 50 Hz and t = 14.5 s); -Ethanol: water (4:1 *v*/*v*) followed by methanol: water (1:1 *v*/*v*).	-Free phenolics 2.7× higher and bound phenolics 1.7× higher versus no PEF	-PEF significantly increased extractable phenolics by permeabilising cell walls. -High electrical energy required; only pretreatment. -Other components unchanged.	[104]
Phenolic Compounds	Microwave-Assisted Extraction (MAE)	DES	-MAE: 1880 W, optimisation of T (50–100 °C), t (5–25 min) and water in DESs (20–70%); -DESs: ChCl: ethylene glycol, ChCl: lactic acid, ChCl: glycerol and ChCl: 1,2-propanediol; -Molar ratio: 1:2 -SLR = 1:10 (*w*/*v*).	-Optimised condition: ChCl:Gly, 100 °C, 13.30 min, water/DES 37.46% (*v*/*v*), 2.89 mg GAE/g BSG (*w*/*v*) -Substantial ferulic and coumaric acids in the extract.	-High dielectric constant and ionic mobility critical; -Risk of degradation at high T; -Possibility of uneven heating in viscous DES.	[26]
Proteins	MAE, UAE, PEF, and Ohmic Heating	Water extraction	-MAE: 50 W, 15 min, 50 °C; -UAE: 20 kHz, 1200 W, 30 °C, 20 min (max 31 °C) -PEF: 4 kV/cm, 10 Hz, 37 °C; -Ohmic heating: 230 V, T ≤ 50 °C, 40 min, electric field 28.7 V/cm. -SLR = 1:10 for all extraction.	-Overall, recovery was 33.7–58.6% -At pH 9: US gave 58.6% protein recovery (24.1 wt%) vs. 37.5% (control); -MAE at pH 9 gave 43.6% recovery; -Ohmic heating: 22.8 wt%, (~52% recovery; -PEF 29.5% recovery, same as control.	-UAE (and MAE/Ohmic) greatly improved protein yield over conventional. -Ultrasonication at mild pH avoids harsh chemicals. -Emulsion stability was poor beyond 2–3 days. -Ohmic heating gave fine emulsions (stability ~2 days) -Proteins targeted alone; phenolics/carbohydrates left behind.	[105]
Proteins	UAE	Alkaline hydrogen peroxide	-Ultrasound 20 kHz, 1200 W, 30 min -Alkaline H_2_O_2_ 3% (pH 12); -SLR = 1:20 (m/v).	-UAE or UAE + H_2_O_2_ raised extraction yield by 70–140% relative to control (recovery ~30%, treatments up to ~73%) -UAE protein recovery (54.33%) increased slightly compared with the control (53.66%).	-UAE + H_2_O_2_ greatly enhanced protein yield and altered techno-functional properties. -Reduced the yields of other biomass like cellulose and hemicellulose.	[106]
Proteins	UAE	Alkaline (NaOH)	-UAE: 150–350 W, treatment time 5–25 min duty cycle 20–100%; -Alkaline: 0.11 M NaOH S:L 1:15 (*w*/*v*).	-Optimised UAE: 250 W, 20 min duty cycle 60% yielded protein recovery 86.16% vs. control (no UAE) 45.71%; -UAE modified protein structures; improved foaming and emulsifying capacity.	-UAE resulted in proteins with low water absorption capacity than the control sample, which might be due to the partial denaturation of protein.	[107]
Proteins	High Intensity Focused Ultrasounds (HIFU)	DES	-DESs screening; -Selected guanidinium chloride: Urea (1:2) for protein extraction optimisation. -HIFU Optimisation: T 25–70 °C, t 2–20 min, BSG 20–130 mg, SLR = 1:250–1:38.5 (*w*/*v*), amplitude 20–80%).	-Optimised conditions: 80%, 20 min, 37 °C, BSG 20 mg; -Yield 13 ± 2 g protein/100 g BSG, recovery of 41 ± 6% of total proteins.	-Medium yield; -Worked with very small sample amount, making scale-up prediction difficult. -DES combination of guanidinium chloride and urea could be evaluated in similar research.	[12]
Proteins	PEF	Water-Alkaline (NaOH)	-Optimisation of PEF parameters: pulses 5000–9000, electric field strength 8–10 kV/cm, and frequency 8–10 Hz; -Alkaline: SLR = 1:10 (*w*/*v*), pH 10, 1 M NaOH, 50 °C, 90 min.	-Optimised condition 5386 pulses, 10 kV/cm, 10 Hz, yielded protein purity of 89.26% compared to 73.00% by alkaline extraction.	-High yield and purity; -Improved amino acid profile, solubility, foaming and emulsifying capacity of protein. -Only protein extraction, leaving cellulose, phenolics in waste stream.	[108]
Proteins	High Hydrostatic Pressure (HHP)	Alkaline	-BSG pellet extraction by centrifugation from wet BSG after mixing with distilled water at 1:20 (dry *w*/*v*), followed by mixing with 0.11 M NaOH at 1:20 ratio, pH 12. -HHP treatment at 0.1, 300, 450 and 600 MPa at 20 °C for 6 min. -Incubation for 1 h at room temperature. -Centrifugation, precipitation.	-Maximum protein yields 131.37 g/kg found at 600 MPa, versus control 104.25 g/kg. -Maximum recovery also at 600 MPa (43.79%) vs. 34.75% for control. -Similar for purity: 56.35% at 600 MPa vs. 51.37% for control. -No significant changes in protein yield, recovery and purity at different HHP treatments.	-Conformational changes and thiol content affected at 600 MPa. -Emulsion activity index decreased after treatment. -Emulsion solubility index, and foaming capacity remained unchanged. -Gelation capacity increased after treatment.	[109]
AXs	UAE	Alkaline	-Alkaline: 1.5 M NaOH, SLR = 1:12, 4 h, 50 °C -UAE: 20 kHz Power 400 W, duty cycle 60%, and extraction time 15 min.	-AXs contents and yield 60.46% and 58.5%, vs. control 56.20% and 38.37%, respectively.	-Reduced Mw and particle size; -Improved solubility and emulsifying properties; -No improvement in freeze-thaw stability.	[101]
AXs	Ultrasound (US), Microwave (MAE), Combined ultrasound and microwave (UMAK)	Alkaline	-Autoclave at 120 °C, Alkaline, US, MAE at different parameters. -SLR = 1:25 (*w*/*v*) -Combined UMAK: 1% NaOH; US 20 kHz + MAE 1000 W; combined 16.3 min total; -Optimised by RSM: US 20 kHz, MAE 1 kW, T 80–90 °C, t 16–60 min.	-UMAK yielded 34.2% arabinoxylan. -US-only alkali gave slightly more total SDF (40.8%) but lower AXs (34.2% vs. 39.7%) and much longer time.	-UMAK sharply reduced extraction time (to ~16 min vs. 60 min) and preserved polysaccharide structure. -Used alkali (not green); -Only fibres targeted, proteins and minor phenolics not recovered; -Shows benefit of hybrid intensification.	[110]

### 5.2. Microwave-Assisted Extraction (MAE)

Microwave-assisted extraction (MAE) utilises dielectric heating to rapidly heat the solvent and biomass matrix, promoting cell-wall disruption and mass transfer of target compounds into the solvent [111]. The literature reports varying MAE efficiencies for BSG bioactives. For example, Birsan et al. evaluated MAE combined with 0.19 M NaOH and compared TPC yields of the BSG mixture (light malt: dark malt, ~9:1 *w*/*w*) with those from alkaline extraction alone and from 60% acetone (in water) extraction [56]. They found that 60% acetone extraction alone yielded 3.85 mg GAE/g BSG dry weight (DW), whereas alkaline extraction yielded 19.20 mg GAE/g BSG DW and MAE combined with 0.75% NaOH yielded 16.94 mg GAE/g BSG DW. Conversely, MAE using DES has demonstrated promising results. López-Linares et al. applied MAE (65 °C and 20 min) with a ChCl:glycerol DES to BSG, achieving phenolic yields up to 3.78 mg GAE/g BSG DW. They achieved a yield over three times that of conventional methanol extraction (MeOH:water, 80:20 *v*/*v*) at the optimised condition (100 °C for 13.30 min). In BSG, MAE-DES has been shown to solubilise hydroxycinnamic acids (ferulic, *p*-coumaric) effectively [26]. In a recent study, Mikkelsen et al. evaluated MAE of BSG protein at 50 W for 15 min, reaching a maximum temperature of 48 °C. At pH 9, they recovered 43.6% of total protein, which was significantly higher than the control (37.5%, water extraction, room temperature, pH 9 for 2 h) [105]. A key limitation of MAE-DES systems is the potential for uneven heating and hot spots, particularly in viscous DESs, which can lead to localised degradation of target compounds and inconsistent extraction activity. Although no BSG-specific study has directly measured local temperature variation, Lorenzo et al. reported hotspot-induced temperature spikes during NADES-assisted extraction of grape pomace and used an ice–water bath to maintain 60–65 °C [112]. Higher MAE temperatures promoted anthocyanin degradation, while prolonged treatment caused matrix carbonisation. Because DES microwave heating depends on solvent composition, viscosity, and dielectric properties, excessive or non-uniform heating may degrade thermolabile compounds and the DES itself [113]. This highlights the need for careful temperature and power control, particularly during scale-up [114].

### 5.3. Pulsed Electric Field (PEF) Assisted Extraction

Pulsed electric field (PEF) technology applies short, high-voltage pulses (typically 1–10 kV/cm) across a treatment chamber with two electrodes, through which the biomass is passed. The resulting transmembrane potential induces electroporation, i.e., the formation of temporary pores in cell membranes, thereby facilitating the release of intracellular compounds [115]. As a non-thermal technique, PEF preserves the integrity of thermolabile biomolecules [116]. This technology has been optimised to boost phenolic yields from BSG. Martín-García et al. reported that pretreating BSG at 2.5 kV/cm and 50 Hz for ~14.5 s increased total free phenolics by ~2.7-fold and bound phenolics by ~1.7-fold compared to extraction without PEF [104]. Paksin et al. optimised PEF-assisted protein extraction from BSG, achieving protein purities of 89.3% and recoveries up to 90% under optimal conditions (5386 pulses, 10 kV/cm, 10 Hz). This process also enhanced essential amino acid content and functional properties, such as solubility and foaming, outperforming alkaline extraction [108]. The non-thermal nature of PEF minimised protein denaturation and the co-extraction of impurities. Similarly, Kumari et al. demonstrated that PEF increased both free phenolic concentration and the release of bound forms [117]. Despite this, PEF treatment requires a conductive medium (often water) and significant electrical energy. As a pretreatment, rather than an extraction method, PEF must be coupled with solvent extraction (often alkaline or DES).

The synergistic enhancement of DES-based extraction by physical intensification techniques is primarily attributed to cell disruption, improved mass transfer, and reduced DES viscosity. These factors also influence the selectivity of target compounds, potentially resulting in co-extraction or loss of non-target biomolecules. However, a critical gap in the current literature is that most studies optimise conditions for a single target, often at the expense of others. For example, phenolic extraction may degrade proteins or AXs, while protein extraction may leave phenolics and AXs in the residue [118]. Thus, while each intensified process offers higher yields for its target, the drawback is incomplete valorisation. This underlines the need for integrated, sequential extraction schemes. The next section will examine multi-target and sequential extraction strategies designed to capture all major BSG biomolecules.

## 6. Impact of Extraction Methods on Structure and Function of BSG Bioactives

The performance of an extraction method should not be judged solely by recovery yield, because extraction is not structurally neutral. In BSG, phenolics are largely cell-wall-bound, proteins are embedded in a fibre-rich matrix and are highly sensitive to pH-driven unfolding. Additionally, AXs are functionally defined by molecular weight, branching pattern, and ferulate-mediated crosslinking. Accordingly, extraction conditions determine not only how much material is released, but also whether the recovered fraction retains the structural features required for antioxidant activity, interfacial functionality, foaming, gelling behaviour, fermentability, or prebiotic action. This distinction is particularly important when comparing DES-based systems with alkali, aqueous alcohols, hydrothermal processes, or enzyme-assisted extraction, because the apparent gain in extractability may come at the cost of altered molecular architecture and lower application value [6,46,54]. Table 3 represents the effects of different extraction technologies on the structural and functional quality of BSG bioactives.

### 6.1. Phenolic Structure, Stability, and Bioactivity

In BSG, phenolics are not simply “present”; they are structurally constrained. Therefore, the main consideration is not merely release from the lignocellulosic matrix, but whether the extraction route preserves the phenolic profile that underpins biological efficacy. Conventional aqueous organic solvents mainly recover free phenolics and typically underperform for esterified hydroxycinnamates. In contrast, alkaline extraction can liberate large amounts of bound ferulic and *p*-coumaric acids but does so under conditions that may also promote oxidation, side reactions, and loss of structural selectivity [56,57]. This is why simply measuring total phenolic content is not enough to make informed decisions. The choice of solvent has been shown to impact not only the antioxidant capacity but also the antimicrobial and antimutagenic properties of BSG phenolic extracts. This confirms that compositional changes during extraction directly influence functionality, rather than merely affecting extract concentration [57].

From that perspective, DES-based extraction is potentially important because its hydrogen-bonding network, tunable polarity, and adjustable acidity can be used to modulate phenolic release under milder bulk conditions than strong alkali. Specifically in BSG, Nkurunziza et al. [11] showed that conventional and DES systems do not release the same cell-wall-bound phenolic fractions, indicating that DESs should be considered profile-shaping solvents rather than just alternative carriers. In BSG, alkaline hydrolysis predominantly releases aglycone ferulic and *p*-coumaric acids and ferulate dimers, whereas a choline chloride: maleic acid DES preferentially recovered a pentose-glycosylated ferulate, demonstrating extraction-dependent differences in phenolic conjugation [11]. More broadly, glycosylation can increase phenolic stability while altering radical-scavenging activity, whereas the stability and degradation of oligomeric phenolics are degree-of-polymerisation dependent; thus, extraction-induced changes in glycosylation, conjugation, or oligomerisation can decouple total phenolic yield from antioxidant performance [107,119]. Likewise, microwave-assisted DES extraction with choline chloride:glycerol improved recovery of phenolic compounds from BSG relative to conventional methanolic systems, while ultrasound-assisted DES extraction further illustrated that pretreatment and DES composition strongly affect the amount and likely the identity of released phenolics [24,26]. However, the present evidence does not yet justify an unqualified claim that DES invariably yields a biologically superior phenolic fraction. Many BSG studies continue to rely on Folin–Ciocalteu and bulk antioxidant assays, which are not specific enough to determine whether improved apparent performance results from better preservation of hydroxycinnamates, an altered free-to-bound phenolic balance, or co-extraction of other reducing compounds [41,44,61]. This review, therefore, argues that DES offers a credible route to functionality-preserving phenolic extraction, but that this claim remains only partially proven until linked phenolic speciation and bioactivity assays are reported together.

A more critical reading of the literature also suggests that DES may prove most advantageous in contexts where oxidative and thermal damage from conventional strategies becomes limiting. While alkaline and hydrothermal methods serve as effective deconstruction tools, their intensity can disrupt the association between yield and biological quality. In contrast, the integration of DES with carefully controlled microwave or ultrasound treatment may enhance mass transfer without necessitating extensive disruption of the macromatrix, thereby providing a more favourable balance between the release of bioactive compounds and preservation of quality. However, this balance is narrow; the very intensification methods that accelerate DES extraction can also create localised overheating, radical formation, or prolonged cavitation exposure that compromise phenolic integrity if poorly controlled [24,26]. The most defensible conclusion, therefore, is not that DES extraction is universally superior, but that it creates a tunable window in which phenolic function may be enhanced at lower chemical severity than alkali, provided that the extracted phenolic profile is directly characterised and not inferred from yield alone.

### 6.2. Protein Structure and Functionality

The extraction method for BSG proteins is critical due to the chemical heterogeneity and prior processing modifications. For example, malting and mashing alter the native state of hordeins and glutelins, and subsequent extraction further determines whether the recovered fraction behaves as a relatively intact protein ingredient, an aggregated denatured concentrate, or a peptide-rich hydrolysate [4]. This matters because protein functionality in food systems depends on structural attributes, including molecular weight distribution, conformational flexibility, hydrophobic group exposure, thiol accessibility, aggregate size, and the balance between solubility and network-forming capacity [120]. Importantly, denaturation induced by extraction is not inherently negative; mild unfolding can enhance emulsification and foaming, while extensive aggregation or hydrolysis may compromise interfacial stability or gelation [4,46,47].

Strongly alkaline extraction is appealing for its high yields but often leads to physicochemical alterations that may impair both interfacial behaviour and nutritional quality. Studies specifically examining BSG proteins indicate that extraction-induced unfolding and aggregation can have mixed effects on functionality. For example, increasing the pH during alkaline extraction promotes protein unfolding and a greater proportion of random-coil structures, while simultaneously improving emulsifying properties. Similarly, moderate heat treatment can induce structural rearrangement and protein aggregation, yet these changes may enhance emulsion stability and foaming capacity [50,121]. During oxidative alkaline H_2_O_2_-assisted ultrasound extraction, an increase in disordered protein structures has also been associated with changes in solubility, water-holding capacity, foaming behaviour, and emulsifying properties [106]. Maillard glycation can further modify the secondary structure of BSG proteins and improve their solubility and emulsifying performance. However, excessive reaction time or severity may promote the formation of Maillard-derived volatile compounds, highlighting the need to carefully control processing conditions [122]. In BSG systems, this can include deamidation, unfolding, aggregate rearrangement, and partial hydrolysis; some of these changes can transiently improve functional performance, but they also reduce control over final ingredient quality. This is illustrated by intensified alkaline systems. For instance, Li et al. reported that ultrasound-assisted alkaline extraction increased protein recovery from 45.71% to 86.16% under optimised conditions and improved foaming and emulsifying properties; however, the same study also observed reduced water absorption capacity, which the authors attributed to partial denaturation [107]. Recent literature highlights that the extraction method is a primary determinant of protein functionality rather than a mere preliminary step. Chin et al. [47] demonstrated that BSG protein functionality depends on the extraction route, while Connolly et al. [64] and Chai and Chen [123] established that the bioactive and techno-functional properties of recovered proteins are closely linked to their processing history. In this context, a high-yield isolate is not necessarily the most valuable product if its emulsifying, foaming, solubility, or hydrolysate bioactivity properties are impaired.

Mikkelsen et al. compared emerging non-solvent-intensive extraction technologies and found that UAE and MAE yielded higher recoveries than the control [105]. However, the emulsions generated from these recovered proteins were not stable beyond two to three days. This finding serves as a critical counterpoint to the prevailing yield-first narrative in protein extraction processes, emphasising that an improvement in protein release does not necessarily correlate with the stability of the resultant ingredient under realistic formulation conditions. Similarly, HHP-assisted alkaline extraction increased protein yield and recovery relative to the control, but Gokulakrishnan et al. [109] observed that although gelation improved following pressure treatment, emulsion activity decreased. Again, extraction-induced conformational changes were functionally ambivalent, not uniformly beneficial [105,109]. This nuanced understanding underscores the complexity of protein extraction and formulation, necessitating a more holistic evaluation of extraction methodologies beyond yield metrics alone.

Enzymatic extraction is often described as gentler, but from a structure–function perspective, it presents a complex dilemma. Proteases and carbohydrases can preserve mild process conditions and generate soluble, potentially bioactive protein fractions, yet they frequently shift the product away from intact functional proteins and towards hydrolysates or peptide mixtures. Connolly et al. [64] and Kriisa et al. [62] showed that enzyme-assisted processing can generate peptide-rich fractions with notable bioactivities and altered sensory and solubility properties, but these products are not functionally equivalent to intact protein concentrates intended for emulsification or network formation. Thus, enzymatic systems should be better viewed as bioconversion routes than as neutral extraction methods.

In this context, DES- and NADES-based systems should be assessed critically as alternative extraction media. Their main advantage is not simply “green extraction” but the ability to weaken protein–polysaccharide interactions under milder pH and temperature conditions than conventional alkali treatment. Wahlström et al. [98] reported that a carboxylate salt–urea aqueous DES achieved a 32% higher recovery of BSG protein than NaOH extraction. However, it is crucial to approach this system with caution. Despite its extraction efficiency, the chemistry is not straightforwardly food-compatible, and purification of residual DES components is a complex downstream requirement. Hadinoto et al. [50] demonstrated that a choline chloride: trehalose NADES recovered 8% more BSG protein with a similar molecular weight distribution and amino acid profile to alkali-extracted protein, but with higher thermal stability and better emulsifying and foaming performance. That finding is more important than the modest absolute difference in yield, suggesting that a milder solvent environment can maintain the balance between protein flexibility and integrity crucial for interfacial functionality. Several DES studies still prioritise recovery over downstream suitability, despite the fact that residual chaotropes or highly interactive DES components can alter protein behaviour. Such alterations may complicate the effective use of the recovered proteins in real-world applications [12,50].

PEF-assisted alkaline extraction provides a valuable benchmark. Paksin et al. [108] reported that PEF-assisted alkaline extraction achieved a protein concentrate that was 13% purer than alkaline extraction alone and also showed improved solubility, foaming, and emulsifying behaviour. The non-thermal nature of PEF treatment likely contributes to these improvements by facilitating matrix permeabilisation while minimising the risk of severe thermal denaturation. However, even here, the system still relies on alkali to achieve extraction, so the functional advantages should be interpreted as the result of a synergistic structural mechanism rather than a stand-alone effect of electroporation. Broadly, protein studies repeatedly show that intensification modulates conformation, and that the desired outcome depends on the final application. For instance, a mildly unfolded protein may be superior for emulsions, whereas a more structured or thiol-reactive fraction may be preferable for gelation [105,108,109].

The key critical conclusion is that DESs are not yet proven to deliver the highest protein recoveries from BSG, but they may already be the most conceptually promising route when the objective is to preserve functional protein architecture rather than simply maximise extraction. This potential is conditional upon several factors: using food-relevant DES compositions, carefully controlling water content and temperature, and ensuring that any residual DES is either removed or analytically accounted for. Additionally, more robust structural evidence is needed. Much of the existing literature infers denaturation or preservation based solely on bulk functionality. A more consistent application of techniques such as SDS–PAGE, intrinsic fluorescence, FTIR/CD, DSC, particle size analysis, ζ-potential measurement, and interfacial rheology would enable stronger conclusions about the relationship between structure and function [12,47,50].

### 6.3. Arabinoxylans (AXs) Structure and Function

Among the three target biomolecule classes, AXs offer perhaps the clearest case for why structure–function analysis is essential. The functionality of AXs is closely tied to several structural factors, including molecular weight, the ratio of arabinose to xylose substitution, ferulate content, degree of aggregation, and the preservation of ferulate-mediated crosslinks during extraction processes. High-molecular-weight, ferulate-containing fractions may be desirable where viscosity, network formation, or encapsulation behaviour is needed; by contrast, lower-molecular-weight fractions or AXs oligosaccharides may be preferable for prebiotic applications and incorporation into beverages or emulsified systems [6,53,54]. Conventional alkali treatment effectively solubilises AXs by disrupting ester linkages within the cell wall. However, this rigorous process can lead to depolymerisation, thereby diminishing the very structural attributes that make AXs valuable as a thickening agent, encapsulation matrix, or fermentable fibre for gut health. Lynch et al. [36] showed that extracted BSG AXs have microbiome-modulation potential, while Liu et al. [101] demonstrated that UAE-produced AXs have lower molecular weight and smaller particle size, but with improved solubility and emulsifying properties. These findings illustrate a crucial point: structural alteration is not inherently negative, but its desirability depends entirely on the target application.

Hydrothermal processing introduces a different trade-off. While it can generate soluble AXs-derived oligosaccharides without added chemicals, increasing the severity of the process often leads to a reduction in molecular weight and, if not carefully managed, can result in the formation of degradation products. Steiner et al. [67] demonstrated that hydrothermal treatment changes the concentration and molecular-weight distribution of BSG AXs and β-glucan. This reinforces the idea that extraction conditions also serve as molecular engineering conditions [54,67].

AX rheology is governed by the combined effects of chain size, branching, and ferulate-mediated cross-linking. Cereal AX fractions with higher molecular weight and ferulic acid content exhibit greater oxidative cross-linking, water binding, and viscoelastic gel strength, while the Ara/Xyl substitution pattern also influences network formation [124]. The most convincing BSG evidence for controlled functional modification currently comes from intensified non-DES systems. Liu et al. [101] achieved increased AXs yield with significantly reduced extraction time by ultrasound-assisted alkaline extraction. Notably, the extracted material exhibited lower molecular weight and smaller particle size, together with improved solubility and enhanced emulsifying and rheological behaviour. These findings are instructive because they suggest that depolymerisation can serve as a beneficial modification under certain conditions, enhancing functional performance in applications reliant on dispersion. Nevertheless, the same study also found no improvement in freeze–thaw stability, implying that the structural changes that favour solubility and emulsification may not favour all hydrocolloid functions equally [101]. Thus, lower molecular weight should be regarded as an application-specific advantage, not as a globally superior arabinoxylan state.

In contrast, DES-enabled AX extraction from BSG remains significantly underdeveloped. In the 2016–2026 literature surveyed here, no BSG-specific primary study was identified that systematically reports a dedicated DES extraction of arabinoxylan from BSG together with structural characterisation. Reviews frequently note that DES chemistry should, in principle, improve fibre accessibility by disrupting H-bonding networks and lignin–carbohydrate interactions, but for BSG AXs, this expectation remains largely mechanistic extrapolation rather than demonstrated evidence [17,54]. The lack of direct evidence is important because the structural effects of DES treatment on arabinoxylan cannot be predicted solely based on yield. Without BSG-specific size-exclusion chromatography with multi-angle light scattering data, monosaccharide compositional analysis, ferulate mapping, and functionality testing, claims that DESs improve AXs functionality remain premature.

Thus, current evidence does not establish DESs as a superior approach for BSG AXs extraction. Rather, their ability to selectively recover AXs while preserving or tailoring their molecular and functional characteristics remains an important unresolved research question that requires direct experimental validation [17,25,80].

## 7. Sustainability Criteria for BSG Valorisation

**Figure 5:**
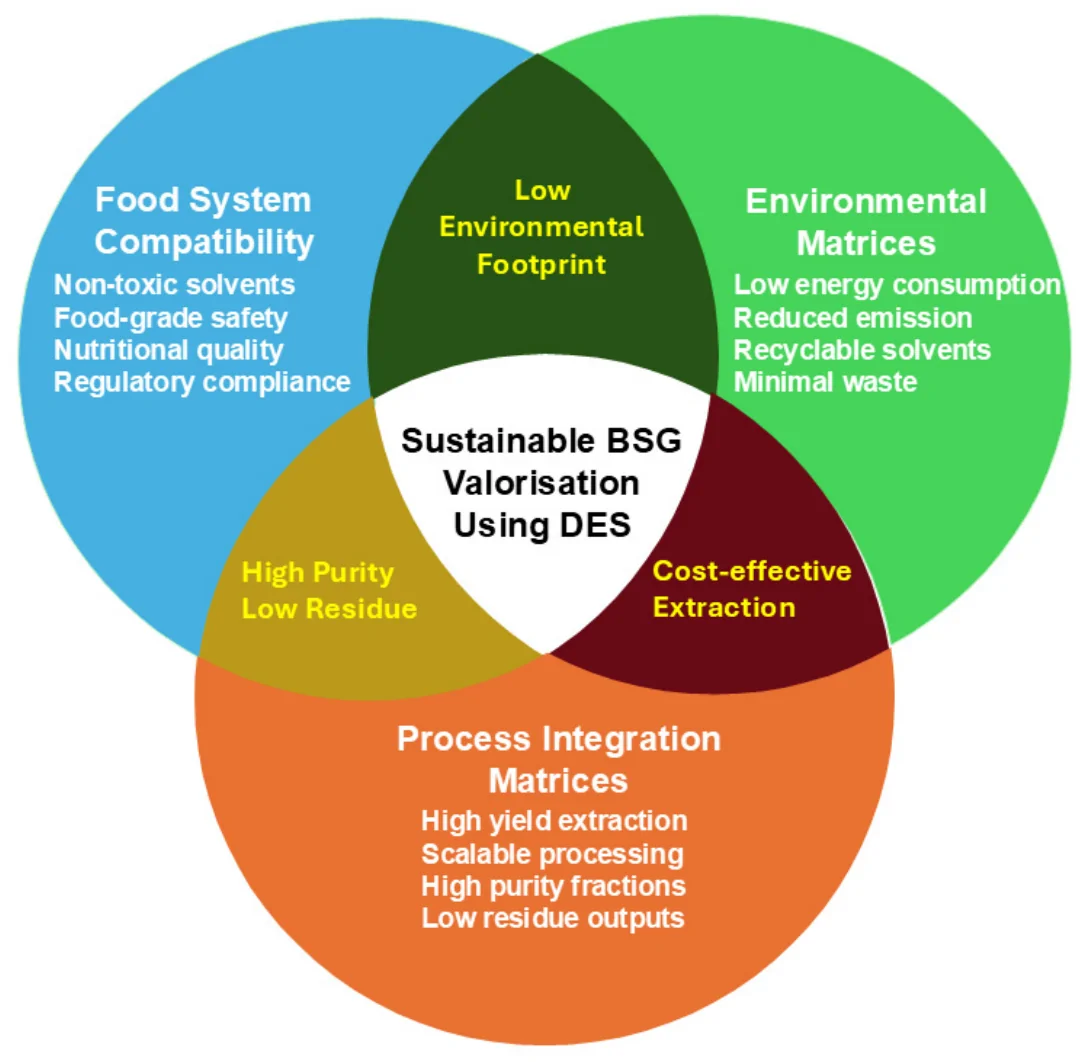
Venn diagram showing the three sustainability pillars and their overlaps for effective valorisation of BSG biomolecules.

Sustainable valorisation of BSG is increasingly recognised as a critical component of the circular bioeconomy, offering opportunities to recover high-value biomolecules, such as phenolics, proteins, and AXs [39]. An effective sustainability framework for sequential BSG valorisation must satisfy three key criteria: environmental impact, process integration, and food system compatibility (Figure 5). Environmental considerations include solvent hazards, specifically toxicity and biodegradability, as well as energy and waste intensity, such as dewatering energy, effluent burden, and solvent composition. Process integration metrics encompass the compatibility of extraction steps, product yield and purity, downstream processing requirements, and robustness to scale or feedstock variability. Food system compatibility involves regulatory approval of solvents, permissible residue levels, and potential sensory or functional effects caused by solvent residues. If any of these criteria are not met, the platform might still extract some products but will struggle to scale sustainably [37].

### 7.1. Environmental Matrices

A fundamental sustainability criterion for any extraction platform is the environmental and human safety profile of the solvent used. The so-called “low volatility” and “bio-derived” solvents do not necessarily mean that a specific solvent is sustainable. Solvent selection must consider human and ecotoxic hazard, exposure potential, persistence, and end-of-life, using structured frameworks [125]. Solvents with no carcinogens, mutagens, or teratogens should be preferred. The biodegradability and recyclability of a solvent must be experimentally measured. Additionally, the solid-to-solvent ratio (g of fresh solvent per kg of BSG) should be considered. It should be noted that solvent recovery processes often require significant volumes of anti-solvents (water, ethanol) and additional energy input for distillation or evaporation, which can offset some of the environmental gains of DES-based extraction [126]. Many DESs use benign components (e.g., choline, sugars, organic acids, polyols) that individually are generally recognised as safe (GRAS) [81]. Still, some high-yielding DES (e.g., guanidine/urea) contain non-GRAS chemicals and would not meet food or environmental safety standards [12].

Energy consumption is a major determinant of the environmental footprint of BSG valorisation. In most bioactive recovery methods, BSG drying is the first sample preparation step [42,51]. Traditional BSG drying to facilitate extraction is highly energy-intensive, with thermal drying requiring at least 2.4 MJ/kg of water removed, making it economically and environmentally unattractive for large-scale operations [127]. Approaches that valorise wet BSG avoid this energy-intensive drying process and often improve overall sustainability [30]. However, wet BSG is highly susceptible to microbial growth. In DES applications, wet BSG may alter the solvent’s properties (water content, viscosity, polarity, etc.). Thermal steps used for hydrothermal extraction, solvent regeneration, and concentration (e.g., evaporation) can erase the benefits of “green solvents.” Therefore, energy consumption per kg of wet BSG and the purified fraction, the time–temperature combination, and whether the process is based on wet or dry BSG should be reported and compared.

The minimisation and management of waste streams are critical for sustainable BSG valorisation. Given the key observation of single components in the current literature, implementing multi-target extraction strategies using a single solvent could be a promising advancement. Furthermore, the use of anti-solvents for product precipitation and solvent recovery often generates substantial volumes of aqueous effluents containing dissolved organics, salts, and residual solvents. These effluents require effective treatment or valorisation approaches. Integrating the valorisation of residual post-extraction biomass within circular biorefinery models is increasingly recognised as a means to maximise resource efficiency and minimise environmental impact.

### 7.2. Process Integration Matrices

A sustainable extraction method for BSG bioactives requires compatibility across all process steps rather than focusing solely on high yield at a single stage. The extraction platform should facilitate the efficient, selective and sequential recovery of multiple high-value fractions from BSG, including phenolics, proteins, and AXs, without excessive cross-contamination or degradation. Phenolic extraction conditions must not irreversibly damage proteins through denaturation or aggregation and must prevent AX degradation through hydrolysis or depolymerisation. DES systems can be a suitable platform for this as they are highly tunable, allowing solvent properties to be tailored for the selective solubilisation of specific biomolecule classes [17,128]. Recent studies on sequential strategies highlight the advantage of milder, integrated steps that balance yields, reduce water and energy use, and support wet BSG processing [31].

The purity of extracted fractions plays a key role in their market value and suitability for food, feed, or material applications. Achieving high purity with minimal downstream processing improves sustainability, as purification steps, such as centrifugation, diafiltration, evaporation, membrane filtration, anti-solvent precipitation, and chromatography, are major contributors to water and energy consumption. For example, traditional alkaline protein extractions often co-solubilise non-protein fractions, whereas selective DES extraction can yield phenolic-rich extracts free of sugars [12]. In contrast, crude ethanolic extracts typically contain sugars, fatty acids, and fibres, necessitating additional separation steps such as centrifugation or filtration [24]. Therefore, minimising and clearly defining downstream purification requirements should be specified to scale up BSG valorisation.

BSG composition varies by barley variety and brewing conditions, so a sustainable platform must tolerate this without frequent re-optimisation. Key robustness indicators include consistent performance on BSG, tolerance to particle-size variability, and predictable solvent viscosity and mass transfer under scale-relevant mixing. High-viscosity solvents may perform well in the lab but can slow mass transfer. Therefore, solvent viscosity (mPa·s) at extraction temperature should be considered. For example, undiluted ChCl:oxalic acid NADES had a viscosity of about 225 mPa·s at 30 °C yet extracted effectively [24]. At scale, this high viscosity may require strong mixing, positive displacement pumps, or a temperature control unit to maintain homogeneous solvent composition [129].

### 7.3. Food System Compatibility

Extraction solvents and process residues must meet strict regulatory standards before BSG-derived fractions are used in food and feed products. When selecting solvents for food ingredients, regulations specify which solvents are allowed and the maximum residue levels. In the European Union, Directive 2009/32/EC covers extraction solvents for foods and ingredients [130]. Food safety authorities note that although most solvents are removed, some unavoidable residues may remain; therefore, safety assessments consider residue limits and exposure [131].

The regulatory status of novel DES formulations, particularly those incorporating less common or synthetic components, may require case-by-case evaluation. Currently, DESs have not been listed as extractant solvents [131]. Consequently, the use of DES in food systems may require evidence demonstrating residual solvent control and safety in the finished fraction. Therefore, physicochemical properties and functionality tests should be conducted on the extracted fractions before use as food ingredients. Even when components are GRAS, residues can alter taste, odour, colour, ionic strength, and protein functionality. Sustainability and food compatibility intersect here: if residues force intensive purification, the environmental footprint of extraction/production increases.

Analytical methods such as ion chromatography, HPLC-CAD, and mass spectrometry are employed to detect and quantify residual solvents and impurities in final products. Regulatory limits are typically set at low ppm levels [132]. Therefore, the quantitative evaluation of solvent residues in each fraction must be demonstrated after extraction.

Despite the potential sustainability advantages of DES-based processing, quantitative environmental evidence for BSG fractionation remains limited. In particular, BSG-specific studies rarely provide standardised information on key factors like energy and water consumption, solvent recovery, material intensity, and waste generation. Consequently, environmental superiority over conventional extraction techniques cannot yet be established solely from extraction performance or solvent characteristics.

Therefore, future comparisons should use cradle-to-gate LCA encompassing BSG stabilisation, solvent production, extraction, purification, solvent recovery, and waste treatment, and report energy demand (MJ or kWh/kg product), water consumption, DES recovery and purity, process mass intensity, and waste generated per unit of recovered fraction. These indicators are important because upstream DES-component production, electricity demand, water use, and downstream purification can substantially alter the overall environmental profile. Indeed, broader biomass-extraction LCAs have shown that DES systems do not invariably outperform water or aqueous ethanol [133,134,135]. Solvent recyclability should also be experimentally verified over repeated cycles. Although DES-based lignocellulosic fractionation has demonstrated recoveries of approximately 92% for ChCl and 96% for ethylene glycol with 98–99% purity, equivalent quantitative recovery data remain limited for BSG-specific processes [136].

## 8. DES Recyclability, Recovery, and Downstream Processing

Recovering and reusing DES after BSG extraction is critical for sustainability. In practice, large solvent volumes are needed for biomass pretreatment; therefore, solvents must be cheap, green, and easy to recover [137]. In current bioactives recovery processes from BSG, a significant amount of solvent remains in the waste stream, making recovery and recycling necessary. The current extraction strategy for BSG bioactives typically includes a cleanup step followed by an extraction stage. Paiva Santiago et al. [24] recommended adding a DES reuse step after BSG phenolics extraction to enhance the environmental and economic value of the extraction process. Isci and Kaltschmitt [126] summarise 44 studies in their review and show that during the extraction of bioactives from different biomass feedstocks, many DESs (especially choline chloride/acid mixtures) can be recycled 4–10 times without major loss of efficiency. Nevertheless, no published BSG-specific study has yet systematically tested DES reuse. Thus, despite claims of “biodegradability and recyclability” for DES, actual reuse in BSG processes remains largely unproven, constituting a clear avenue for further investigation.

Common strategies for recovering DES after extraction include physical and chemical separation methods. For example, vacuum evaporation using a rotary evaporator is often used to remove water and other volatile co-solvents, thereby concentrating the DES [138]. Membrane-based methods, such as ultrafiltration or dialysis, effectively separate small DES molecules from larger solutes. Other options include adding an anti-solvent (such as water, ethanol or acetone) to precipitate out the extracted compounds while retaining DES in solution, which is then separated by filtration or decanting [126,137]. For example, Mamilla et al. added water and acetone to a spent ChCl–oxalic acid DES to separate solids and recovered approximately 75% of the DES by precipitation [139]. Published studies on DES recovery list techniques such as freeze-drying, electrodialysis, or short-path distillation, each presenting trade-offs in energy consumption and operational complexity [126,137,138].

Once extraction is done, downstream processing must isolate the target biomolecules (phenolics, proteins, AXs) from the DES. For protein-rich extracts, standard strategies include isoelectric precipitation or ultrafiltration. In one DES-extraction technique, the BSG was first defatted, then treated with a NaAcO:urea DES. The liquid extract was filtered and dialysed (3.5 kDa cutoff) to separate proteins from the DES [98]. They achieved ~80% protein yield with a ChCl:urea DES from BSG but noted a drawback: residual DES in the protein isolate. This underscores the need for careful purification. Any remaining DES (or its components, such as urea) must be removed by washing, dialysis, or reprecipitation, etc., to meet food-grade standards.

In summary, research on DES recycling and downstream purification in BSG valorisation is still in its early stages. Available studies indicate that solvent recovery through evaporation, membrane separation, and similar methods can reduce operational costs, while several biomass-based investigations demonstrate the feasibility of multi-cycle DES reuse [16,137]. But critical gaps remain: most BSG-DES studies do not report actual recycling experiments, and effective strategies for removing residual DES from the extracted products are still lacking. Addressing these will be essential for feasible, circular BSG biorefineries.

## 9. Multi-Target Bioactives Extraction: Current Status and Limitations

BSG valorisation has traditionally focused on extraction of a singular component. For instance, many studies isolate proteins or phenolics separately, often under harsh conditions, leaving the fibrous matrix largely unexploited [30]. The concept of multi-target extraction involves the stepwise or integrated recovery of multiple valuable fractions, such as phenolics, proteins, and AXs, from a single biomass feedstock, thereby maximising resource efficiency and minimising waste. In the context of BSG, this approach aligns with the principles of biorefinery and zero-waste valorisation [55,140].

Following the methodology of a patented work [141], a four-step alkaline/water fractionation of wet BSG enabled the recovery of proteins (~65%), cellulose (~85%), AXs (~90%) and ~50% of lignin under mild conditions [30]. This process also included ATR-FTIR spectroscopy to monitor fractionation in real time. Although the authors reported that the resulting fractions were high-value and near-zero-waste, an alkaline solution was used as the solvent, and detailed downstream purification procedures were not fully described. In a recent study, the same research group reported recovery of approximately 65% of the protein and 91% of gluco-, xylo-, and arabino-oligosaccharides from wet BSG. They achieved this using Alcalase in a mild alkaline environment (pH 8.5), which also allowed them to recover phenolics (11 mg GAE/g dry BSG) during the protein extraction step. The yields were high, and contaminants were minimal; however, the method relied on sequential enzymatic hydrolysis combined with high-temperature hydrothermal reactors [31]. This proved expensive given the high cost of the enzymes, energy, and infrastructure, along with the potential thermal degradation of sensitive BSG bioactives. de Crane d’Heysselaer et al. [142] performed a two-step acid–alkali treatment involving an aqueous 0.127 M H_2_SO_4_ solution followed by 0.75 M NaOH at 120 °C. This process achieved approximately 80% recovery of hemicelluloses, 68% of cellulose, and 70% of pure lignin from dried BSG [142]. The study demonstrated that conventional acid/alkali fractionation effectively separates fibre fractions. The authors also identified a significant challenge: BSG batches exhibited considerable variability in protein (13–23%) and hemicellulose (9–23%) content. This variability arises from the intrinsic differences in the chemical composition of BSG, which are influenced by the various cereal varieties selected by brewers. Such variability complicates the standardisation of processes [142]. Roy et al. [18] reported a DES-containing multi-target approach that combined NADES with subcritical-water hydrolysis and obtained BSG hydrolysates containing proteins, sugars, flavonoids, and individual phenolic compounds. Although this strategy demonstrates simultaneous recovery of chemically diverse bioactives, the compounds were recovered as a mixed hydrolysate rather than as discrete purified fractions. Consequently, the process reduces the emphasis on target-specific extraction but transfers a substantial challenge to downstream fractionation and product purification [18].

These pioneering multi-target studies have demonstrated that sequential extraction is feasible and can be productive, but they also reveal significant limitations. Firstly, no standardised workflows exist. Each study uses different solvents, enzymes, or temperatures, preventing direct comparisons of methods or the adoption of best practices across laboratories. For example, Belardi et al. [30] used only water and mild alkali, whereas de Crane d’Heysselaer et al. [142] applied strong heat and acids. Secondly, carry-over effects remain largely uninvestigated. Each extraction step could alter the remaining material, for example, by altering pH or partially degrading polymers, but existing reports do not systematically quantify the loss or retention of each component at each stage. For instance, Belardi et al. [31] observed that alkaline protein extraction co-extracted phenolics but did not report the fraction of fibre remaining for subsequent steps. Without mass balances or residue analyses, the true independence of these steps remains unclear.

Comparison of these strategies reveals two conceptually different routes to multi-target BSG valorisation. Sequential fractionation processes recover compositionally distinct streams in successive steps. For example, mild water/alkali processing has enabled the recovery of proteins, AXs, cellulose, and lignin, whereas enzymatic extraction followed by hydrothermal treatment has produced protein, phenolic, and oligosaccharide fractions [30,31]. In contrast, intensified approaches such as NADES-modified subcritical-water hydrolysis simultaneously release proteins, sugars, and phenolics into a common hydrolysate, increasing overall biomass utilisation but shifting the separation burden downstream [18]. Thus, sequential processing generally offers greater fraction selectivity but requires multiple unit operations, whereas simultaneous co-extraction can reduce the number of extraction steps but produces more compositionally complex streams requiring subsequent purification.

The aforementioned works demonstrate that current methods of BSG bioactives extraction have both economic and technical drawbacks. Enzymatic and hydrothermal approaches produce pure products [31] but come with enzyme costs and high energy use. Harsh chemical treatments [142] work well but need corrosion-resistant reactors and generate substantial neutralisation by-products, as the acidic or alkaline reagents must be neutralised before disposal, producing additional salt-rich wastewater. So far, all processes have been tested only at the lab scale, and their scalability and cost-effectiveness are still unknown. Many successful extractions also require drying beforehand or the use of expensive solvents. No stepwise extraction method fully avoids these issues, so a completely sustainable process has yet to be achieved. Future research should focus on developing appropriate workflows and on understanding how each variable interacts with the others.

## 10. Challenges and Future Directions

**Figure 6:**
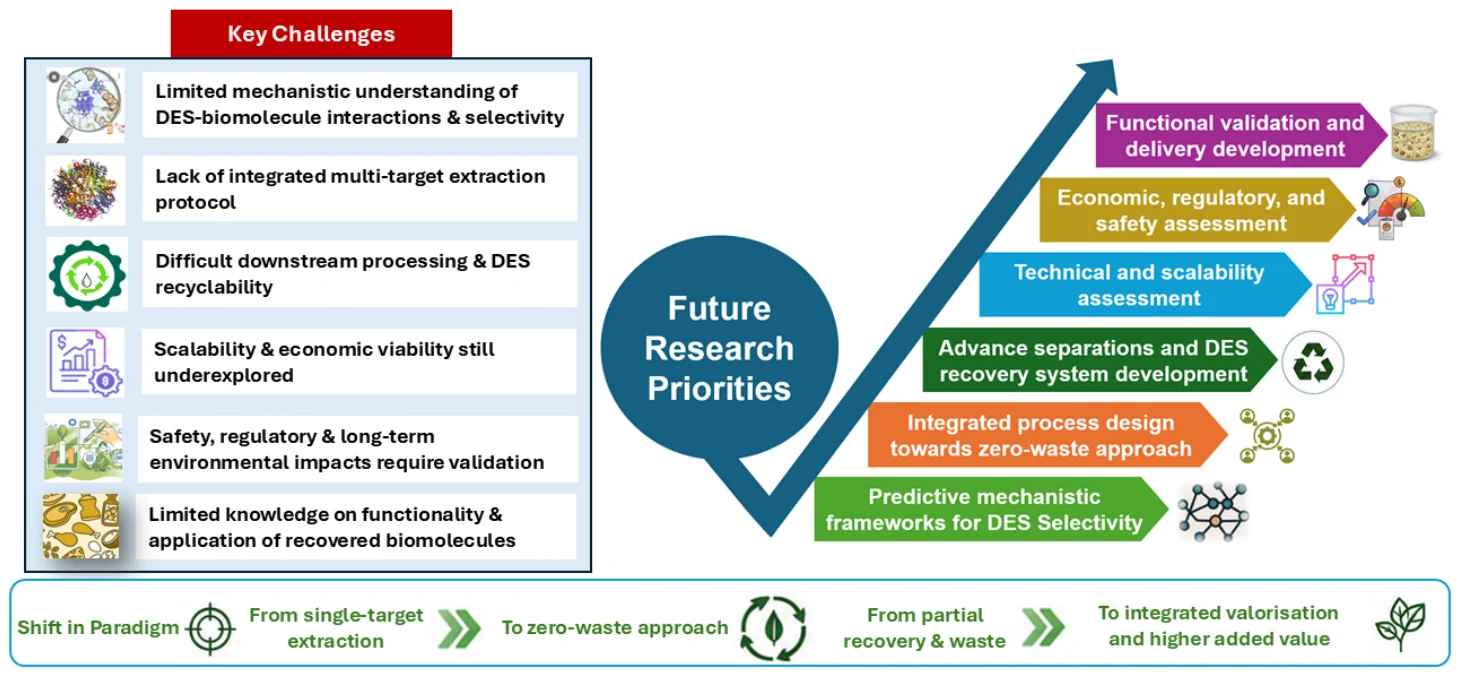
Existing challenges in current research on BSG bioactive extraction techniques and prospects for future research.

BSG valorisation through the extraction of high-value biomolecules has garnered significant attention over the past decade. As discussed in the previous sections, many recent studies have reported the efficient recovery of proteins, phenolics, and AXs from BSG using DES and alkaline solvents, combined with microwave- and ultrasound-assisted extraction techniques. Despite these advances, significant research gaps and technical challenges remain, impeding the development of integrated, scalable, and commercially viable BSG biorefineries (Figure 6).

A significant research limitation is that DES selection for BSG fractionation is mostly empirical, with little mechanistic understanding of selectivity among phenolics, proteins, and hemicellulose. BSG phenolic acids are mainly cell wall bound, so their extraction under acidic DES or hydrothermal conditions reflects both solvation and matrix breakdown, not just solvent partitioning [97]. Many studies rely on bulk measurements that fail to distinguish between free and bound phenolics, co-extracted carbohydrates that affect properties, or chemical changes that alter bioactivity and downstream separability [12,97]. Protein extraction also relies heavily on alkaline or chaotropic DES, where pH and denaturation effects dominate, making it hard to separate DES-specific actions from typical alkaline extraction, and complicating the preservation of protein functionality for food uses [12].

A further limitation is the insufficient molecular-level characterisation of recovered BSG bioactives. Structural preservation or modification is frequently inferred from extraction yield or functional measurements rather than demonstrated directly. Future studies should therefore integrate advanced structural analyses with functional assays. For phenolics, targeted or untargeted LC–MS/MS profiling is needed to distinguish changes in individual free and bound compounds. Protein studies should combine SDS–PAGE and molecular-weight profiling with FTIR, circular dichroism, and DSC to assess aggregation, secondary structure, and thermal stability. Similarly, AXs characterisation should include molecular-weight distribution, arabinose-to-xylose ratio, substitution patterns, and ferulate/diferulate analysis. Such complementary characterisation would provide stronger evidence for extraction–structure–function relationships and enable more reliable comparison among conventional, DES-based, and intensified extraction strategies.

Despite promising laboratory-scale results, the scalability and economic viability of DES-based BSG valorisation remain largely unproven [143]. Most published studies are limited to small-scale, batch processes, with little attention to process optimisation, energy consumption, and cost analysis. High solvent-to-solid ratios, long extraction times, and the requirement for specialised equipment, such as ultrasound or microwave intensification, further hinder scale-up [16,143]. Although many DES components are relatively inexpensive and readily available, techno-economic assessments remain limited, and existing analyses indicate that challenges associated with solvent recovery, DES recyclability, downstream purification, and energy-intensive processing may substantially increase overall operational costs [144]. In particular, the low vapour pressure and non-volatile nature of DES complicate solvent removal and recovery, posing additional technological challenges during downstream processing [83]. These limitations may offset the environmental and safety advantages of DES compared to conventional solvents. For instance, the minimum selling price of protein extracted from BSG via optimised microwave-assisted alkaline extraction exceeds market prices significantly unless co-extraction of other bioactives and process integration are implemented [145]. Therefore, these techno-economic factors must be considered to enable effective valorisation of BSG bioactives using DES. In short, the field lacks a comprehensive, predictive framework for DES-biomolecule interactions in BSG matrices. This gap hampers the rational selection and optimisation of DES systems for selective, high-yield extraction of target biomolecules.

It has been well documented that DES and NADES for BSG primarily optimise the extraction of individual compounds, such as phenolics or proteins, or, at most, protein–phenolic co-extraction [97,98,123]. Residual fibres, particularly AXs, are often undervalued and discarded as waste streams. Furthermore, when AX extraction is the primary objective, proteins and phenolics are frequently overlooked [110]. AX-focused studies highlight the need to preserve macromolecular integrity. For instance, solid-state fermentation-based extraction yields high-quality AXs, while hydrothermal processes result in increased yields but reduced M_w_ alongside an increased risk of undesirable by-products [36]. Although sequential processing can achieve multi-fraction recovery, current approaches are mainly water/alkali-based, and true DES-enabled sequential strategies remain undeveloped [10,36]. A sequential extraction strategy employing DES has the potential to address these limitations by utilising its tunable solvation properties to selectively target specific bioactive classes in stages, while preserving the structural integrity of remaining fractions for subsequent recovery.

Over the past decade, improvements in extraction yields have increased more rapidly than efficiency in downstream processing. The non-volatility and high viscosity of DES complicate solvent removal, selective precipitation, and recycling, often increasing overall water and energy demand due to extensive dilution, washing, and multi-step purification [81]. Although several separation strategies have been reported, they are rarely demonstrated for BSG biomolecule triads (phenolics, proteins, and AXs). For instance, cellulose ultrafiltration can purify spent DES [146], and an ultrafiltration–diafiltration–nanofiltration process has demonstrated both high polymer separation and DES recovery, indicating that membrane-assisted recycling is feasible if viscosity is managed [147]. However, DES recyclability remains constrained by the accumulation of degradation products, such as organic acid derivatives, sugar dehydration products (e.g., furfural and hydroxymethylfurfural), and dissolved lignin fragments, which increase viscosity, alter pH, and reduce delignification efficiency [148]. Collectively, high viscosity and associated mass-transfer limitations, difficult solvent recovery, downstream purification requirements, formulation-dependent toxicity and regulatory uncertainty, and the scarcity of techno-economic validation currently prevent DESs from being regarded as an established superior alternative for BSG valorisation.

Recent advances in process intensification, such as continuous-flow extraction and membrane-assisted solvent recovery, highlight the potential for scalable DES-based biorefineries; however, these systems remain underexplored for BSG applications. Rigorous validation under industrially relevant conditions, including complex feedstock variability and spent-DES chemistries, is essential to translate these conceptual frameworks into viable, circular bioprocesses.

Current studies provide limited attention to the functional application of recovered biomolecules. Specifically, research on the stability of extracted AXs, proteins, and phenolics and their potential for structured delivery systems is lacking. Therefore, future studies should investigate their functionality, bioaccessibility, and compatibility in food and nutraceutical formulations to enable the development of high-value applications from BSG-derived bioactives.

Regulatory and safety considerations present additional hurdles, especially for food and nutraceutical applications. While DESs and NADESs are often promoted as green and safe solvents, their environmental and toxicological profiles remain poorly understood [149]. Although most DESs are readily biodegradable and exhibit low acute toxicity, their long-term ecological impacts, especially under large-scale use and disposal conditions, warrant further investigation [144,149]. The literature emphasises that toxicity depends on DES constituents, molar ratios, pH, and solute interactions, while mechanistic discussions highlight how high viscosity can bias diffusion-based toxicity assays [150]. Addressing these regulatory challenges is critical for translating laboratory advances into industrial practice and for ensuring the safety and acceptance of DES-derived BSG products.

Future research should therefore prioritise predictive, mechanistic frameworks for DES–biomolecule selectivity alongside validation of solvent recovery and food safety pathways to support scalable BSG biorefineries. Emphasis must shift beyond maximising extraction yields of individual components toward integrated BSG valorisation, where extraction, separation, and purification are co-designed. This includes incorporating phase partitioning, anti-solvent precipitation, membrane fractionation (e.g., ultrafiltration/nanofiltration), and DES-compatible chromatographic strategies. Particular attention should be given to DES recyclability under realistic process conditions, including the accumulation of impurities, viscosity changes, and shifts in solvent physicochemical properties after repeated cycles.

## 11. Conclusions

Brewer’s spent grain (BSG) is an abundant lignocellulosic by-product with significant potential as a sustainable source of phenolics, proteins, and AXs. This review highlights the growing role of deep eutectic solvents (DESs) as green extraction media that have shown potential to improve extraction selectivity and process sustainability under selected conditions. Emerging process-intensification approaches, particularly ultrasound- and microwave-assisted extraction, further demonstrate the potential to enhance recovery efficiency while reducing processing time and energy demand.

Despite these advances, most current research focuses on single-target extraction, while integrated sequential recovery of multiple BSG bioactives remains underdeveloped. Furthermore, limited mechanistic understanding of DES–biomolecule interactions, inadequate solvent recovery strategies, and the lack of industrial-scale validation continue to constrain commercial implementation. Future research should therefore prioritise rational DES design for integrated isolation of all valuable BSG biomolecules, solvent recyclability, and comprehensive downstream processing, including membrane fractionation, anti-solvent precipitation, and DES-compatible purification systems. Greater attention is also needed to evaluate the functionality, structural integrity, stability, and application potential of recovered biomolecules in food and nutraceutical systems.

Overall, DES-assisted BSG valorisation represents a promising pathway for transforming a low-value brewing by-product into a sustainable source of high-value functional ingredients for food and nutraceutical applications.

## Figures and Tables

**Table 1:** Summary of extraction approaches for BSG bioactives (proteins, phenolic compounds, and arabinoxylans), using traditional and emerging extraction methods.

Extraction Method	Extraction Conditions	Protein Yield	Phenolic Yield	Arabinoxylans Yield	Key Notes /Drawbacks	References
Solvent Extraction	-Aqueous ethanol, acetone, or methanol (50–80% *v*/*v*), -Solid–liquid ratio (SLR) = 1:10–1:20 -Temperature: 30–60 °C, -Time: 1–24 h	-Low; mostly insoluble, extracted mainly hordein, only ~15–20% of total protein.	-Yields free phenolics only; e.g., acetone/ethanol extraction recovered ferulic acid < 0.01% of BSG (very low). -Optimising solvent mix (e.g., 50–70% aqueous ethanol) gives ~0.1–2.0 mg GAE/g TPC.	-Not effective. Solvent extraction does not solubilise fibre; -AXs remain in residue.	-Simple and cheap. -Extracts mostly free compounds. -Low yield for proteins and bound phenolics. -Requires large solvent volume and long time (up to 24 h). -Environmental and safety concerns with organic solvents.	[16,27,41,44,47,56,57]
Alkaline Hydrolysis	-Mainly NaOH 0.1–4 M (pH ~9–13), 25–80 °C, SLR = 1:10–1:30, -Time: 0.25–4 h (often 1–3 cycles)	-Very high; solubilises protein. -NaOH 0.1 M at pH 12 yielded ~50–70% protein.	-Releases bound phenolics. -TPC: ~0.5–4.7 mg/g -Ferulic acid up to ~4.7 mg/g -Yields ferulic acid up to ~1% of BSG (5× increase over no alkali).	-Partial solubilisation of hemicellulose. -Strong alkali (pH > 12) -Extracted ~60% of AXs as soluble fibre (often precipitated with alcohol).	-Highly effective for breaking cell-wall linkages; inexpensive reagents. -But can degrade sugars and denature proteins (loss of functionality) if too harsh. -Co-extracts lignin and oligos -Requires neutralisation of waste liquor.	[16,27,29,30,47,49,51,58,59]
Acid Hydrolysis	-Dilute H_2_SO_4_ or HCl 0.1–2%; 100–160 °C (or autoclave); SLR = 1:20; Time: 0.5–4 h.	-Yield: ~0–10% (protein recovery very low; yields amino acids). -Severe conditions hydrolyse proteins to amino acids, not usually used for protein extraction (more for hydrolysates).	-Negligible, ~0–0.5 mg/g (often degraded). -Not used to extract intact phenolics–tends to destroy them.	-Yield: ~0–10% intact; high monomer (xylose) release -Dilute H_2_SO_4_ yielded high sugar release but degraded AX structure (suitable for fermentation, not for AX product).	-Good for converting polymers to fermentable sugars. -Not used for intact protein or phenolic recovery. -Produces sugars and degradation products (furfural, HMF).	[27,47,60]
Enzymatic Extraction	-Proteases (Alcalase, Flavourzyme) at pH 7–9, 40–60 °C, 1–24 h; -BSG to distilled water ratio 1:10–1:20 (*w*/*v*), -Carbohydrases (xylanase, feruloyl esterase) for AX/phenolics	-Yield: ~40–85% (peptides/hydrolysates); -Proteins retain more functionality due to mild conditions.	-Phenolics: ~0.5–3 mg/g (bound phenolics released by feruloyl esterases); -Carbohydrases (xylanase, ferulic acid esterase) release bound phenolics. -Enzyme-treated BSG showed higher phenolic content and antioxidant activity than untreated. -Often requires subsequent solvent to collect released phenolics.	-Yield: ~10–60%. -Xylanases can solubilise arabinoxylan oligomers. -Complete AX recovery requires multi-enzyme (xylanase + arabinofuranosidase) cocktails.	-Mild and specific. -Proteases solubilise proteins (preserving function); -Carbohydrases (xylanase/feruloyl esterase) release bound phenolics and partial AXs. -Slower and costlier than chemical methods. -Typically slower and may need preprocessing (grinding, etc.).	[16,31,42,48,61,62,63,64,65]
Subcritical Water Extraction (SWE, Hydrothermal)	-Water at 120–200 °C, 10–25 bar, 5–60 min (autohydrolysis); -BSG to distilled water ratio 1:8–1:33 (*w*/*v*), -Residence time and Temperature control severity	-High; -Hot water (160–180 °C) can solubilise 50–80% of proteins (often as peptides).	-Moderate; releases bound phenolics as part of autohydrolysis. -Some ferulic and *p*-coumaric acids co-extract when hemicellulose is hydrolysed. -Phenolic yield less studied.	-Very high for oligos; -Autohydrolysis freed ~91% of hemicellulose as soluble arabino-xylooligosaccharides. -AX oligomers obtained without sugar degradation when optimised.	-Uses only water (no toxic chemicals). -Efficiently fractionates fibre into oligos. -Needs pressure vessels and careful control to avoid degradation (furfurals). -Typically yields a mixed hydrolysate (requires downstream separation of sugars, phenolics, etc.).	[16,60,66,67,68,69,70]

**Table 3:** Effects of different extraction technologies on the structure–function relationships of BSG bioactives, extraction techniques, and key gaps.

Target Fraction	Extraction Method /Solvent	Structural Changes Induced	Functional Changes Observed	Key Reporting Gaps	Key References
Phenolics	Aqueous-organic extraction (ethanol, methanol, acetone)	-Mainly extracts free and weakly bound phenolics; -limited cleavage of ester-linked ferulates.	-Moderate antioxidant activity; -limited recovery of bound phenolics.	-Often reports TPC only; -limited LC-MS characterisation.	[41,44]
	Alkaline extraction (NaOH, KOH)	-Ester bond cleavage; release of ferulic and *p*-coumaric acids; -partial oxidation possible.	-Increased antioxidant capacity due to liberation of bound phenolics.	-Rare assessment of oxidation products and structural integrity.	[11,43,56]
	Hydrothermal/ subcritical water extraction	-Hydrolysis of cell-wall matrix; -release of bound phenolics; -possible thermal degradation at high severity.	-Improved antioxidant activity; -generation of mixed hydrolysates.	-Limited compound-specific profiling; -degradation products rarely quantified.	[31,67,96]
	Enzyme-assisted extraction	-Selective cleavage of cell-wall linkages; -preservation of phenolic structures.	-Enhanced bioaccessibility and antioxidant activity.	-Limited techno-economic assessment.	[31,61]
	DES extraction	-Disruption of hydrogen-bond networks; -controlled release of bound phenolics.	-Improved antioxidant retention and phenolic preservation.	-Residual solvent effects poorly studied.	[11,97]
	DES-UAE	-Enhanced cell disruption and mass transfer; -minimal thermal damage	-Increased phenolic recovery and antioxidant activity.	-Ultrasound energy density often unreported.	[24,25]
	DES-MAE	-Rapid matrix disruption; -accelerated release of hydroxycinnamates.	-Increased extraction efficiency and antioxidant activity.	-Thermal stability of individual phenolics rarely evaluated	[25,26]
	PEF-assisted extraction	-Electroporation increases accessibility without chemical modification.	-1.7–2.7-fold increase in phenolic recovery.	-Mechanistic studies still limited; -lack of scale-up data.	[104]
Proteins	Alkaline extraction	-Protein unfolding, deamidation, -aggregation, partial hydrolysis.	-Increased solubility and recovery; -variable emulsification.	-Structural characterisation often incomplete.	[47,49,71]
	Enzyme-assisted extraction	-Generation of peptides and hydrolysates.	-Enhanced digestibility and bioactivity; -almost 100% water soluble; -emulsion activity index (EAI) 83 m^2^/g protein; -emulsion activity index (ESI) ≈ 35 min; -comparable to alkaline/ethanolic isolates.	-Difficult comparison with isolate-based studies.	[62,64]
	DES extraction	-Reduced denaturation; -preservation of molecular weight distribution.	-Improved foaming and emulsifying properties; -higher thermal stability.	-Residual DES removal rarely quantified.	[50,98]
	DES-UAE	-Controlled unfolding; -increased exposure of hydrophobic groups.	-Improved extraction and interfacial functionality; -EAI: conventional 40.53 vs. UAE 51.72 m^2^/g; -ESI: conventional 53.62% vs. UAE 67.63%; -Foaming capacity: conventional 65% vs. UAE 81%; -Foam stability: conventional 64.29% vs. USE 78.57%, after 120 min.	-Lack of scale-up data.	[12]
	UAE-assisted alkaline extraction	-Reduced aggregate size; -partial denaturation; -α-helix decreased by 4.76%, while β-sheet increased by 5.83%; -changes indicate partial unfolding/reorganisation rather than complete hydrolysis.	-Improved foaming and emulsification; -reduced water-holding capacity; -Foam stability: UAE 107.33% vs. 84.78% for conventional extraction.	-Few studies correlate structure with functionality.	[106,107]
	HHP-assisted alkaline extraction	-Conformational rearrangement; -altered aggregation behaviour; -α-helix increased by 8%, while β-sheet reduced by 8% for 300 MPa than control; -partial unfolding/reorganisation rather than complete hydrolysis.	-Improved gelation; reduced emulsion activity; -EAI: control 159.0 vs. 450 MPa 135.3 m^2^/g; -Foaming capacity: no significant change; -Foam stability: control 28.2% vs. 600 MPa 73.3%.	-Limited molecular characterisation.	[109]
	PEF-assisted alkaline extraction	-Enhanced permeability with less thermal damage.	-Increased purity, solubility, foaming, and emulsification;-Emulsifying ability: Alkaline 96.69%, PEF 99.58%; -Emulsion stability: 90.98% vs. 91.20%; -Foamability: Alkaline 7.84%, PEF 30.86%; -Foam stability: 68.89% vs. 79.37%.	-Mechanisms require further study.	[108]
AXs	Alkaline extraction	-De-esterification; cleavage of lignin-carbohydrate complexes.	-Increased solubility and yield.	-Feruloylation rarely quantified.	[54,101]
	Hydrothermal extraction	-Depolymerisation; -reduced molecular weight.	-Increased soluble fibre and oligosaccharide formation.	-Limited rheological characterisation.	[67]
	Enzyme-assisted extraction	-Controlled depolymerisation; -AXOS generation.	-Enhanced prebiotic potential.	-Structure–function relationships poorly understood.	[6]
	UAE-assisted alkaline extraction	-Reduced molecular weight and particle size.	-Improved solubility, emulsification, and rheological properties.	-Limited application-specific evaluations.	[101]
	Combined UAE–MAE	-Accelerated depolymerisation and release.	-Enhanced soluble fibre recovery.	-Molecular-weight distributions not consistently reported.	[110]

## Data Availability

No new data were created or analysed in this study. Data sharing is not applicable to this article.

## Abbreviations

The following abbreviations are used in this manuscript:

ATR–FTIR	Attenuated total reflectance–Fourier-transform infrared spectroscopy
AXOS	Arabinoxylo-oligosaccharides
AXs	Arabinoxylans
BSG	Brewer’s spent grain
ChCl	Choline chloride
DES/DESs	Deep eutectic solvent/deep eutectic solvents
DSC	Differential scanning calorimetry
DW	Dry weight
EFSA	European Food Safety Authority
FA	Ferulic acid
FTIR	Fourier-transform infrared spectroscopy
GAE	Gallic acid equivalent
GRAS	Generally recognised as safe
HBA	Hydrogen-bond acceptor
HBD	Hydrogen-bond donor
HDES	Hydrophobic deep eutectic solvent
HHP	High hydrostatic pressure
HIFU	High-intensity focused ultrasound
HMF	5-Hydroxymethylfurfural
HPLC–CAD	High-performance liquid chromatography–charged aerosol detection
LA	Lactic acid
LC–MS	Liquid chromatography–mass spectrometry
LC–MS/MS	Liquid chromatography–tandem mass spectrometry
MAE	Microwave-assisted extraction
MeOH	Methanol
Mw	Molecular weight
NADES/NADESs	Natural deep eutectic solvent/natural deep eutectic solvents
NaOH	Sodium hydroxide
OA	Oxalic acid
PEF	Pulsed electric field
RSM	Response surface methodology
SDF	Soluble dietary fibre
SDS–PAGE	Sodium dodecyl sulfate–polyacrylamide gel electrophoresis
SLR	Solid-to-liquid ratio
SWE	Subcritical water extraction
TPC	Total phenolic content
UAE	Ultrasound-assisted extraction
UMAK	Ultrasound- and microwave-assisted alkali extraction
US	Ultrasound
